# Cancer Drug Delivery with Nanoparticles and Biomolecules: Stimuli-Responsive, Theranostic, and AI-Guided Approaches

**DOI:** 10.3390/ma19173729

**Published:** 2026-09-01

**Authors:** Chiara Boncristiani, Francesca Baldassarre, Khadija Eddahaoui, Giuseppe Ciccarella, Viviana Vergaro

**Affiliations:** 1 Experimental Medicine Department, University of Salento, Via Monteroni, 73100 Lecce, Italy; chiara.boncristiani@unisalento.it; 2 Institute of Nanotechnology (CNR NANOTEC), Consiglio Nazionale delle Ricerche, S. P. 6 Lecce-Monteroni SNC, 73100 Lecce, Italy; francesca.baldassarre@unisalento.it (F.B.); giuseppe.ciccarella@unisalento.it (G.C.); 3 Tecnomed Puglia—Technopole for Precision Medicine (Biotech Lecce Hub), c/o Ecotekne Campus, via Monteroni, 73100 Lecce, Italy; 4 National Interuniversity Consortium of Materials Science and Technology (INSTM), Via G. Giusti 9, 50121 Florence, Italy; khadija.eddahaoui@unisalento.it; 5 Laboratory of Physical Chemistry of Applied Materials LCPMA, Faculty of Sciences Ben M’Sick, Hassan II University of Casablanca, B.P. 7955 Bd Cdt Driss El Harti, Casablanca 20702, Morocco; 6 Department of Biological and Environmental Sciences and Technologies (DiSTeBA), University of Salento, S. P. 6 Lecce-Monteroni SNC, 73100 Lecce, Italy

**Keywords:** nanoparticles, biomolecule drug delivery, natural compounds, nucleic acid therapeutics, peptide-functionalized nanocarriers, tumor microenvironment, AI-guided drug design, precision oncology

## Abstract

**Highlights:**

- Critically compares nanoplatforms through structure–property–function relationships.
- Identifies the engineering trade-offs limiting clinical translation of cancer nanomedicines.
- Unifies smart, stimuli-responsive, and theranostic strategies for precision cancer therapy.
- Evaluates advanced 3D tumor models as predictive tools for nanoparticle performance.
- Explores AI-guided nanocarrier design and biomolecule-based therapeutics for next-generation cancer drug delivery.

**Abstract:**

Cancer remains a major global health challenge, and the limitations of conventional therapies, including systemic toxicity, drug resistance, and poor tumor selectivity, continue to drive the development of advanced nanomedicine strategies. In this context, nanocarriers offer promising opportunities to improve pharmacokinetics, enhance tumor accumulation, and enable controlled or stimuli-responsive drug release. Among them, inorganic nanoparticles (NPs) have gained considerable attention because of their structural stability, tunable surface chemistry, and multifunctional capabilities. Their performance depends on a structure–property–function relationship in which composition, morphology, porosity, degradability, and surface characteristics strongly influence interactions at the nano–bio interface. This review examines the main classes of nanoplatforms currently explored for cancer therapy, including inorganic, polymeric, lipid-based, and hybrid organic–inorganic systems. Particular attention is given to the trade-offs that define each platform in terms of loading capacity, biodegradability, multifunctionality, and translational potential. The discussion also highlights the role of predictive biological models, emphasizing that 3D spheroids, organoids, and organ-on-chip systems provide more realistic insights than conventional 2D assays for evaluating tumor penetration and microenvironment-responsive delivery. In addition, the review considers emerging directions in AI-guided nanoparticle engineerization design and natural-compound-based nanomedicines, both of which are expanding the therapeutic landscape. Overall, the field is moving toward more integrated, application-specific, and clinically translatable nanomedicine platforms capable of addressing the complex biological barriers of cancer treatment.

## 1. Introduction

Cancer remains a major global health burden, and current therapies are still limited by systemic toxicity, drug resistance, and insufficient tumor selectivity [1,2,3]. Nanomedicine offers a strategy to improve pharmacokinetics, enhance tumor accumulation, and enable controlled or stimuli-responsive drug release. Among nanocarriers, inorganic NPs are of particular interest because of their structural stability, tunable surface chemistry, and multifunctional properties. Their biomedical performance is governed by a structure–property–function relationship in which composition, morphology, porosity, surface chemistry, and degradability determine interactions at the nano–bio interface and, consequently, therapeutic outcome [4,5]. Inorganic platforms can also support imaging, magnetic guidance, photothermal conversion, photocatalysis, and microenvironment-responsive delivery within a single construct [6,7,8]. However, no nanoplatform is universally optimal. Each material involves a specific trade-off between loading capacity, biodegradability, multifunctionality, and translational feasibility [3,9,10]. This is evident when comparing calcium carbonate, iron oxide, gold, silver, mesoporous silica, titanium dioxide, and hydroxyapatite systems, which differ in degradability, imaging capability, cargo loading, and responsiveness to endogenous or external stimuli [11]. Polymeric NPs and lipid-based systems further broaden the design space. Their main advantage lies in formulation flexibility, as size, charge, degradation rate, surface functionality, and release kinetics can be tuned through polymer composition and architecture [12]. In parallel, hybrid organic–inorganic nanostructures combine the functional robustness of inorganic components with the biocompatibility and processing advantages of organic matrices. These systems are increasingly used to overcome limitations in drug stability, circulation time, and tumor selectivity [13]. The relevance of these systems also depends on predictive biological evaluation. Conventional 2D assays remain useful for initial screening, but 3D spheroids, organoids, and organ-on-chip platforms better reproduce tumor heterogeneity and transport barriers and therefore provide more informative translational models. This issue is particularly important for nanocarriers designed to penetrate dense tumor tissue or respond to the tumor microenvironment [14,15]. This review also addresses AI-guided nanoparticle design and natural-compound-based nanomedicines. AI is increasingly used for formulation optimization and property prediction, while natural products remain attractive therapeutic cargos despite limitations in solubility, stability, and bioavailability. Overall, the field is moving toward integrated, application-specific, and clinically translatable nanomedicine.

### 1.1. Rationale for Nanoparticle-Based Drug Delivery

Conventional cytotoxic chemotherapy suffers from a set of interrelated pharmacological limitations that motivate the shift toward nanoparticle-based delivery discussed throughout this review. First, most small-molecule chemotherapeutics lack intrinsic tumor selectivity and distribute according to their physicochemical properties rather than disease location, producing dose-limiting toxicities in rapidly dividing normal tissues (bone marrow, gastrointestinal epithelium, hair follicles) that constrain the maximum tolerated dose and, consequently, therapeutic efficacy [16,17]. Second, several clinically important agents (e.g., paclitaxel, docetaxel, camptothecins) are poorly water-soluble, historically requiring formulation in solubilizing excipients (Cremophor EL, polysorbate 80) that themselves cause hypersensitivity reactions and neurotoxicity [16,18,19,20,21]. Third, rapid renal or hepatic clearance and short plasma half-lives necessitate frequent, high-dose administration, further amplifying systemic exposure [17]. Fourth, multidrug resistance (MDR), mediated principally by ATP-binding-cassette efflux transporters such as P-glycoprotein, progressively reduces intracellular drug accumulation and is a leading cause of treatment failure and relapse [22,23,24]. Nanocarriers address these limitations concurrently rather than individually: encapsulation improves aqueous solubility without toxic excipients, modifies pharmacokinetics and biodistribution to reduce off-target exposure, enables passive and/or active tumor accumulation (Section 2 and Section 7.2.2), and can co-deliver MDR-reversing agents or promote endocytic rather than passive-diffusion cellular entry, partially bypassing efflux-transporter-mediated resistance. This shift in rationale—from treating the tumor as a diffusion-accessible mass to treating it as a biologically and physically heterogeneous barrier (Section 1.2)—underlies the platform-by-platform comparisons developed throughout this review.

### 1.2. The Tumor Microenvironment as a Barrier to Drug Delivery

The tumor microenvironment (TME) is not a passive host tissue but an actively hostile, heterogeneous ecosystem that constitutes the principal physical and biological barrier to effective drug delivery, independent of the delivery vehicle chosen. Structurally, tumor vasculature is architecturally abnormal, characterized by irregular, tortuous, and hyperpermeable vessels with discontinuous basement membranes, alongside regions of poor or absent perfusion; this heterogeneous vascularization produces spatially variable drug and nanocarrier access even within a single tumor mass. Functionally, impaired lymphatic drainage elevates interstitial fluid pressure (IFP), which opposes the pressure gradient needed for convective transport of therapeutics from the vasculature into the tumor interstitium and instead confines delivery largely to diffusion-limited transport. This is compounded by a dense, desmoplastic extracellular matrix (ECM), rich in collagen and hyaluronic acid and particularly pronounced in stroma-rich tumors such as pancreatic ductal adenocarcinoma, which further restricts penetration of both free drugs and nanocarriers (Section 7.1). Metabolically, poor perfusion generates regions of hypoxia and acidosis (Section 4.1), which alter drug efficacy and select for more aggressive, treatment-resistant cellular phenotypes, while also providing endogenous triggers exploitable for stimuli-responsive release (Section 4). Finally, the TME hosts an immunosuppressive milieu, including regulatory T cells, tumor-associated macrophages, and myeloid-derived suppressor cells, that limits both endogenous anti-tumor immunity and the efficacy of immunotherapeutic nanocarriers (Section 4.4). Conventional chemotherapy and even simple, non-targeted nanocarriers relying solely on passive EPR-mediated accumulation address the vascular-abnormality component of this barrier only partially and do not, by themselves, overcome elevated IFP, dense ECM, or the immunosuppressive milieu; this is precisely why the stimuli-responsive, actively targeted, and immunomodulatory nanocarrier strategies developed throughout Section 4, Section 5 and Section 6 of this review have become central to the field [25,26,27,28,29,30].

## 2. Inorganic NPs for Oncological Drug Delivery

Inorganic nanoparticle performance is better explained by a structure–property–function framework, in which composition, morphology, porosity, surface chemistry, and degradability jointly determine biological behavior and therapeutic potential [4,5]. This structural design enables distinct therapeutic functions but also imposes specific translational constraints, making platform selection application-dependent rather than governed by synthesis simplicity or loading capacity alone [3,6,7,8,9,10,11]. Table 1 lists inorganic NPs used as drug delivery agents for cancer therapy. NPs can penetrate tissue and accumulate at target sites, protect encapsulated drugs from degradation, enable sustained release, and evade immune recognition to prolong circulation, improving efficacy while reducing toxicity [1,12,31,32]. Superparamagnetic iron oxide NPs (SPIONs) are among the most extensively studied biomedical nanomaterials, valued for their magnetic properties, biocompatibility, and chemical stability [33,34]. Their magnetite/maghemite core enables remote magnetic manipulation, T2-weighted MRI contrast, and magnetic hyperthermia within a single theranostic platform [35,36,37,38,39], but their limited drug-loading capacity relative to porous carriers and rapid MPS clearance, driven largely by opsonization and protein-corona formation and typically resulting in predominant hepatosplenic sequestration (Section 10.2), often requires extensive surface engineering. Gold NPs (AuNPs) derive their versatility from surface plasmon resonance, generated by collective electron oscillation upon light irradiation [39]. Anisotropic shapes (nanorods, nanoshells, nanostars) shift resonance into the NIR region, enabling photothermal conversion, photoacoustic imaging, and radiosensitization [4,40], while robust Au-S chemistry allows facile conjugation of ligands, polymers and nucleic acids [4,41,42]. Poor biodegradability and long-term tissue accumulation, driven largely by protein-corona-mediated opsonization and subsequent mononuclear phagocyte system (MPS) uptake in the liver and spleen (Section 10.2), remain the main translational barriers [43,44]. Calcium carbonate NPs (CaCO_3_ NPs) are intrinsically responsive to the tumor microenvironment: preferential dissolution under mild acidity enables selective drug release while buffering extracellular pH and generating CO_2_, aiding drug penetration and potentially enhancing ultrasound imaging [45,46,47]. Unlike AuNPs or SPIONs, they exploit endogenous rather than external triggers, and their high biodegradability distinguishes them from many inorganic systems [48,49,50], positioning them as biodegradable, multifunctional theranostic platforms. Silver NPs (AgNPs) act through a mechanism in which controlled release of Ag^+^ ions induces oxidative stress, providing broad-spectrum antimicrobial and anticancer activity while narrowing the therapeutic window. For this reason, surface functionalization is often designed to tune dissolution kinetics and ion bioavailability. AgNPs can also exhibit a localized surface plasmon resonance (LSPR) typically around ~400–450 nm, a property exploited for SERS, colorimetric sensing, and bioimaging; however, the intensity and spectral position of the plasmonic response depend strongly on nanoparticle size, geometry, degree of aggregation, dielectric environment, surface chemistry, and excitation wavelength, so comparisons with other materials (e.g., AuNPs) should be qualified by these parameters and experimental conditions [51,52,53]. Mesoporous silica NPs (MSNs) provide features advantageous for delivery, notably ordered mesopores that allow tunable, high-capacity encapsulation of drugs, proteins, nucleic acids, and imaging agents, and extensive silanol chemistry that facilitates functionalization with stimuli-responsive gatekeepers and targeting ligands [26,39,40,41,42]. Calling MSNs “ideal” is not appropriate without specifying relevant parameters: performance is governed by pore architecture, particle size, surface functionalization, and the biological environment. Benefits such as high loading and surface versatility must be explicitly balanced against practical and safety limitations, including biodegradation kinetics, protein-corona formation and consequent hepatosplenic sequestration, long-term biodistribution, potential toxicity, and scale-up challenges (Section 10.2); these factors require case-by-case evaluation and further in vivo studies [41,54,55,56,57]. Titanium dioxide NPs (TiO_2_ NPs) occupy an intermediate operational space: their semiconducting structure can generate reactive oxygen species under UV/visible excitation, which is useful for photodynamic and antimicrobial applications, but efficacy depends on parameters such as crystalline phase, particle size, surface coatings, and excitation wavelength. Consequently, practical limitations—including the need for light activation, concerns about persistence and phototoxicity, and application-specific constraints—have influenced their translation into clinical and large-scale uses in antimicrobial coatings, photodynamic therapy, biosensing, and tissue engineering [58,59]. Hydroxyapatite NPs (HAp NPs) closely resemble bone mineral, giving strong osteoconductivity and skeletal affinity; their biodegradability and calcium/phosphate release make them attractive for bone-targeted therapy and regenerative scaffolds, though use remains largely confined to orthopedic/dental applications [60,61,62].

No inorganic nanoplatform is universally optimal: each balances multifunctionality, loading capacity, biodegradability, imaging, and clinical feasibility differently [1,63]. Progress will likely depend on engineering application-specific platforms matched to the intended biological function rather than identifying a single superior material [64].

**Table 1:** Summary of inorganic NPs and their synthesis, properties, and biomedical applications.

Inorganic NPs	Synthesis	Surface Functionalization	Key Properties & Functions	Biomedical Applications	Advantages	Limitations	Ref.
Superparamagnetic iron oxide (SPIONs)	Co-Precipitation, thermal decomposition, microemulsion, laser-assisted	PEG, dextran, PVP, PLGA, silica, albumin, HA, FA, targeting ligands	Superparamagnetic core for MRI/hyperthermia; coating dictates stability and biological fate	MRI-guided delivery, magnetic targeting, hyperthermia, theranostics	Remote magnetic control, MRI contrast, multifunctionality, biocompatibility	Aggregation/oxidation if uncoated, rapid MPS clearance, lower drug loading	[65]
Gold (AuNPs)	Turkevich, Brust–Schiffrin, seed-mediated, microfluidics	Thiolated PEG, antibodies, RGD peptides, HA, FA, DNA/RNA	LSPR enables photothermal conversion and imaging; robust Au–S bioconjugation	Photothermal therapy, targeted delivery, CT/photoacoustic imaging, biosensing	Outstanding optical properties, easy functionalization, high chemical stability	Poor biodegradability, tissue accumulation, possible accelerated clearance post-PEGylation	[66]
Calcium carbonate (CaCO_3_)	Precipitation, biomimetic mineralization, continuous-flow, spray drying	PEG, lipids, cell membranes, HA, FA, antibodies, APTES/MPTES	Porous, biodegradable matrix; acid-triggered dissolution enables pH-responsive release and tumor buffering	pH-responsive delivery, gene editing, US imaging, theranostics	High biocompatibility, biodegradability, high loading, scalable	Premature dissolution without coating, limited mechanical robustness	[67]
Silver (AgNPs)	Chemical reduction, polyol, laser ablation, green synthesis	Citrate, PVP, PEG, chitosan, alginate, collagen, targeting ligands	Controlled Ag^+^ release and ROS generation; plasmonic properties for SERS	Antimicrobial coatings, wound healing, SERS, anticancer therapy	Broad-spectrum antimicrobial activity, strong plasmonic response, simple synthesis	Dose-dependent cytotoxicity, oxidative instability, limited therapeutic window.	[65]
Mesoporous silica (MSNs)	Sol–gel (Stöber), surfactant-templated, microemulsion	PEG, PEI, lipid bilayers, gatekeepers, DNA, targeting ligands	Ordered mesoporous structure provides extreme loading capacity and stimuli-responsive release	Controlled/gene delivery, combination therapy, imaging	Very high loading capacity; tunable pore size; excellent chemical versatility	Slow biodegradation, potential silica accumulation, complex scale-up	[66]
Titanium dioxide (TiO_2_)	Sol–gel, hydrothermal/solvothermal, flame synthesis	PEG, silica, polymers, HA, metal/non-metal dopants	Semiconductor photocatalysis generates ROS under light irradiation	Photodynamic therapy, antimicrobial coatings, biosensing	High photostability, inexpensive production, multifunctionality	UV dependence (unless doped), ROS toxicity, limited tissue penetration	[68]
Hydroxyapatite (HAp)	Wet precipitation, hydrothermal, biomimetic mineralization	Collagen, gelatin, chitosan, PEG, ionic substitutions	Bone-like composition gives intrinsic osteoconductivity and sustained release	Bone regeneration, osteosarcoma therapy, bone-targeted delivery	Excellent osteointegration, intrinsic bioactivity, biodegradability	Restricted mostly to skeletal applications, low mechanical strength	[69]

### 2.1. Synthetic Strategies for Inorganic Nanoplatforms

Nanoparticle physicochemical properties (size, morphology, crystallinity, surface chemistry, porosity, colloidal stability) are dictated by synthetic strategy and directly govern biological interactions and therapeutic performance. Preparation methods are broadly physical, chemical, or biological, each with distinct trade-offs in reproducibility, scalability, purity, and process complexity [70,71,72,73].

Physical methods (laser ablation, vapor deposition, combustion/flame synthesis) avoid extensive chemical reactions, yielding highly crystalline, pure NPs, but are limited by costly instrumentation, high energy use, and low throughput [74,75,76].

Chemical synthesis (precipitation, sol-gel, thermal decomposition, microemulsion, hydrothermal, reduction reactions) is the most versatile and widely adopted strategy, offering precise control of nucleation/growth and compatibility with large-scale manufacturing, though organic solvents/surfactants require extensive purification before biomedical use [75,77].

Green/biomimetic synthesis exploits microorganisms, plant extracts, or biomolecules as reducing/stabilizing/templating agents, minimizing hazardous chemicals and improving biocompatibility, though reproducibility and scale-up remain challenges for clinical translation [71,78].

Continuous manufacturing (continuous-flow reactors, spray-drying, microfluidics) is increasingly replacing batch processes, offering better control of reaction kinetics and heat/mass transfer, narrower size distributions, and higher batch-to-batch reproducibility [73,79].

### 2.2. Material-Specific Synthetic Strategies

Although synthesis principles are broadly shared, each inorganic material requires specific preparation strategies tailored to its physicochemical properties and biomedical performance.

CaCO_3_ NPs are mainly produced via controlled precipitation and biomimetic mineralization, with continuous-flow and spray-drying emerging as scalable, reproducible alternatives offering good control over polymorphism and morphology, which govern degradation kinetics and drug release [80,81].

SPIONs are most commonly produced by co-precipitation (simple, scalable) or thermal decomposition (gold standard for crystalline, monodisperse particles); physical techniques (laser-assisted synthesis, vapor deposition) give high purity but are less suited to industrial production [33,82].

AuNPs are mainly synthesized by chemical reduction: Turkevich citrate reduction for spherical particles, Brust–Schiffrin for thiol-protected nanoclusters, and seed-mediated growth for anisotropic shapes (nanorods, nanostars, nanoshells) [83]; microfluidic synthesis now enables reproducible continuous-flow production [84].

AgNPs are primarily obtained by chemical reduction of silver salts, with polyol synthesis, laser ablation, and green routes also used [85,86,87]; reducing agent and stabilizer choice strongly influences ion release, size, and stability, determining both antimicrobial efficacy and cytotoxicity [88].

MSNs are produced through surfactant-assisted sol-gel hydrolysis/condensation of alkoxysilanes [89]; sacrificial templating enables precise control of pore architecture and surface area, favoring high-capacity, stimuli-responsive drug loading [90].

TiO_2_ NPs are mainly prepared via sol-gel or hydrothermal routes for controlled crystal phase and morphology [91]; flame-based methods enable continuous large-scale production, while doping extends photocatalytic activity into the visible range [92].

HAp NPs are synthesized by wet chemical precipitation or biomimetic mineralization under mild aqueous conditions, reproducing native bone composition while allowing cation substitution tuning of crystallinity and osteoconductivity [60,93,94].

## 3. Polymeric NPs and Hybrid Organic–Inorganic Systems

### 3.1. Biodegradable Polymeric NPs

Biodegradable polymeric NPs (BPNPs) represent the earliest and most extensively investigated generation of nanocarriers developed for cancer drug delivery. Their emergence marked a paradigm shift from conventional systemic chemotherapy, characterized by poor pharmacokinetic profiles, nonspecific biodistribution, and severe dose-limiting toxicities. Biodegradation is not merely a mechanism for NPs elimination but an engineering parameter that directly controls therapeutic performance. BPNPs improve cancer therapy by simultaneously modulating drug stability, pharmacokinetics, biodistribution, and release kinetics rather than acting solely as passive drug carriers [95].

**Figure 1:**
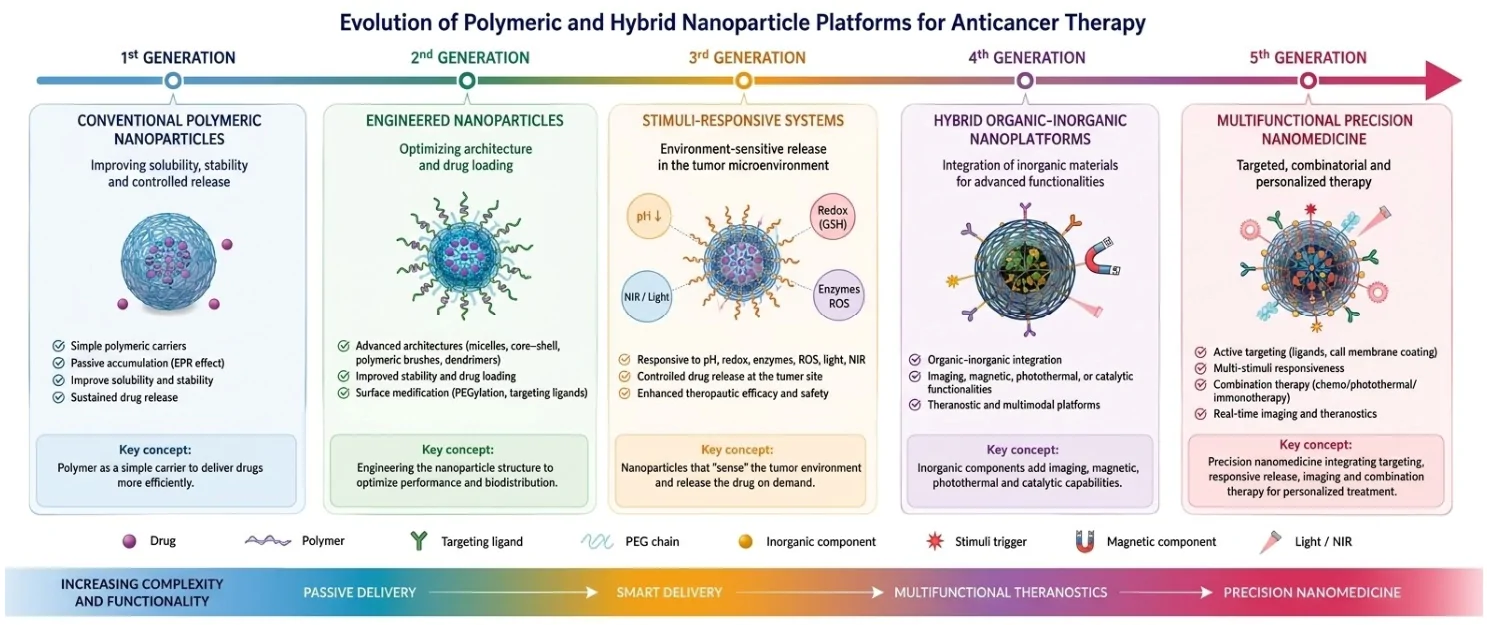
Evolution of BPNPs and hybrid NPs for anticancer therapy. *During the preparation of this figure, the author(s) used Google Gemini to generate the graphical illustration depicting the generational evolution of biodegradable polymeric and hybrid nanoparticles, based on the authors’ original concept, structural layout, and scientific content. The authors have reviewed and edited the output and take full responsibility for the content of this figure*.

The review of the polymeric and hybrid NPs literature has highlighted the technological evolution of these systems, that has been represented in Figure 1.

This evolution can be described as the progression from the conventional, simple first-generation carriers to the more sophisticated, engineered second-generation and stimuli-responsive third-generation systems. Finally, the latest developments in the field of hybrid and multifunctional nanoplatforms are indicated as the fourth- and fifth-generation systems. It should be noted that this generational classification is the authors’ own simplified organizational framework for structuring this review, rather than a formally established or universally adopted taxonomy in the nanomedicine literature. In particular, the boundary between the “hybrid” (fourth-generation) and “theranostic” (fifth-generation) categories is not strictly exclusive, since many hybrid organic–inorganic constructs are themselves inherently stimuli-responsive and/or theranostic; the two categories are therefore best understood as overlapping design trends that frequently co-occur within the same construct, rather than as sequential, mutually exclusive developmental stages.

The design philosophy underlying first-generation BPNPs was relatively straightforward: to create biocompatible carriers able to encapsulate therapeutic agents, protect them from premature degradation, and release them in a controlled manner. Their versatility allowed researchers to modulate particle size, morphology, surface chemistry, degradation kinetics, and drug release profiles through rational polymer engineering, for the efficient entrapment of a wide array of therapeutic payloads.

Natural polymers (chitosan, gelatin, dextran, inulin) resemble ECM/endogenous macromolecules, offering excellent biocompatibility for hydrophobic drug transport, though batch variability and unpredictable degradation complicate reproducibility [96].

These natural polymers share excellent biocompatibility but differ in mechanical strength and degradation predictability, requiring careful selection according to the intended application [97].

To address these limitations, considerable research has focused on synthetic biodegradable polymers, which offer greater control over molecular architecture, physicochemical properties, and manufacturing reproducibility. Among them, poly(lactic-co-glycolic acid) (PLGA) has become the benchmark material for polymeric drug delivery, reflecting its long history of use in several FDA- and EMA-approved pharmaceutical and medical products, its tunable degradation rate, and extensive clinical experience [98]. Upon hydrolytic degradation, PLGA releases its two monomeric building blocks, which follow distinct metabolic routes: lactic acid is converted to pyruvate and enters the tricarboxylic acid (Krebs) cycle, whereas glycolic acid is either excreted unchanged via the kidney or oxidized to glyoxylate before entering central metabolism; both routes ultimately yield carbon dioxide and water [98,99]. This well-characterized, low-toxicity degradation profile minimizes systemic toxicity and avoids the long-term tissue accumulation often associated with inorganic materials.

The versatility of PLGA enables the encapsulation of a broad range of therapeutic agents, including hydrophobic drugs, hydrophilic compounds, proteins, nucleic acids, and combination therapies. Furthermore, the physicochemical attributes of these polymeric matrices are highly tunable by strategically modulating parameters such as the molecular weight, the lactide-to-glycolide molar ratio, and the terminal group chemistry, providing precise control over degradation kinetics and drug release behavior. Such flexibility has supported the development of nanoparticle formulations carrying clinically relevant chemotherapeutics, including cisplatin, docetaxel, paclitaxel, and numerous natural anticancer compounds, demonstrating improved pharmacokinetic profiles compared with free drug administration [100,101].

These carriers have demonstrated high encapsulation efficiency not only for conventional hydrophobic and hydrophilic chemotherapeutics but also for fragile molecular machinery, including gene modulators like Cyclin-Dependent Kinase (CDK) inhibitors, which are protected from enzymatic degradation in the bloodstream [98].

While early research focused predominantly on conventional synthetic chemotherapeutics, the paradigm has rapidly expanded toward green nanomedicine, where biodegradable polymers are uniquely leveraged to unlock the therapeutic potential of poorly soluble phytochemicals. This platform has successfully revitalized the use of bioactive natural compounds and phytochemicals, such as curcumin, paclitaxel, resveratrol, and quercetin, which have historically demonstrated profound multi-targeted antitumor, antioxidant, and anti-inflammatory activities in vitro [16].

Biodegradable polymeric matrices—most notably PLGA, polycaprolactone (PCL), and polylactic acid (PLA)—offer an elegant solution to these pharmacological limitations by physically entrapping these sensitive phytochemicals within their hydrophobic cores, significantly improving their stability and bioavailability [102]. The integration of phytochemical-loaded polymeric NPs into oncology also opens up highly promising avenues for synergistic combination therapies, as discussed later in this review [103].

Despite these advantages, the clinical translation of bare polymeric matrices is often hampered by rapid immune clearance, necessitating advanced surface modifications to optimize their in vivo performance. Therefore, the biological performance of BPNPs is dictated not only by polymer composition but also by the precise engineering of their physicochemical characteristics to overcome severe limitations due to systemic physiological barriers.

PEGylation is widely applied to reduce opsonization and MPS clearance after intravenous administration, extending circulation time and passive tumor accumulation. The free PEG termini can also be functionalized with targeting ligands (antibodies, peptides), bridging passive accumulation with receptor-mediated delivery and improving the pharmacokinetic and safety profile of BPNPs [104].

Active ligands are used to increase selectivity, retention, uptake, and accumulation at tumor sites while reducing off-target exposure [105,106]. It is important to distinguish two conceptually and experimentally distinct effects often conflated under the term “active targeting”: (i) an increase in the total amount of nanocarrier reaching and remaining within the tumor mass (whole-tumor accumulation), governed principally by EPR, circulation half-life, and vascular/stromal barriers rather than by the presence of a targeting ligand per se, and (ii) an increase in binding to, and internalization by, target-positive cells once the nanocarrier has already extravasated into the tumor, which is the effect most directly attributable to ligand–receptor recognition. Because ligand modification acts principally at this second stage, it can substantially improve cellular uptake, intracellular drug delivery, and therapeutic efficacy without necessarily increasing the total injected dose reaching the tumor as measured by whole-tumor biodistribution assays. This distinction should be kept in mind when interpreting studies reporting improved “targeting” based on cellular uptake data alone.

Ligand targeting remains mostly preclinical, still constrained by membrane penetration, specificity, and translational feasibility [107,108,109]. Folate is the most mature ligand strategy, though transferrin and aptamers are gaining traction [110]. Beyond pharmacokinetics, polymeric matrices are evolving toward multifunctionality (MDR reversal, TME immunomodulation, combination immunotherapy) [111], driving a second generation of nanocarriers, exemplified by self-assembled polymeric micelles that increase aqueous drug solubility 10- to 5000-fold [112,113].

This structural degradation, triggered by internal (pH, enzymes) or external (light, temperature, magnetic field) cues, marks the transition from conventional to stimuli-responsive (“third-generation”) nanoplatforms, discussed in the next section [112].

In this section, a series of recent studies are presented to illustrate the evolution of BPNPs from passive drug carriers to multifunctional nanoplatforms.

The study by Gomes-Da-Silva et al. adopts a more comprehensive translational approach by integrating in vitro, in vivo, and computational investigations. The authors exploit the well-established biocompatibility and biodegradability of PCL NPs to improve the antitumor potential of lamivudine (used in the treatment of HIV and hepatitis B infections). By combining controlled drug release, biodistribution analysis, molecular imaging through radiolabeling, and in silico target validation within a single experimental framework, the study broadens the functional role of BPNPs from passive delivery vehicles to translational platforms supporting both therapeutic optimization and mechanistic drug development [114].

Al-Nakashli et al. demonstrate an important shift in the design philosophy of polymeric micelles, showing that drug loading should be considered a critical engineering parameter rather than merely a formulation outcome [115]. Using self-assembled Poly(N-(2-hydroxypropyl) methacrylamide) PHPMA-based polymeric micelles loaded with ellipticine, the authors showed that while maintaining an unchanged polymer composition, increasing drug loading significantly enhances intracellular drug accumulation and anticancer efficacy in both conventional 2D and 3D cell cultures.

BPNPs are evolving from passive carriers into multifunctional therapeutic platforms. The study on the α-tocopheryl succinate-loaded polymeric nanovehicle based on a methacrylic derivative of hydroxychloroquine (HCQ) introduces an innovative strategy in which the polymeric matrix itself actively contributes to the therapeutic effect. Rather than functioning solely as a structural scaffold, the polymer is chemically engineered to incorporate HCQ, transforming the nanocarrier into a pharmacologically active component capable of synergizing with the encapsulated anticancer agent [116].

Verma et al. have recently proposed micellar amphiphilic nicotinamide-bPEI conjugates, designed to act as dual-purpose gene delivery vectors and antiproliferative agents. Furthermore, using vitamin derivatives to engineer self-assembled micelles offers an alternative to fully synthetic, petroleum-based polymers [117].

Gao et al. developed Pluronic P123 (P123)-based polymeric micelles decorated with alendronate (ALN) and cancer-specific phage protein DMPGTVLP (DP-8) for synergistic dual-ligand architecture designed to specifically target breast cancer and the complex bone metastasis microenvironment. The site-specific release of loaded doxorubicin (DOX) has been obtained, also exploiting the pH-sensitive feature of the P123 [118].

A novel active-targeting platform has been designed to improve the treatment of hepatocellular carcinoma by encapsulating lenvatinib (a gold-standard multikinase inhibitor) within engineered polyaspartamide-based NPs with PEG segments terminal-functionalized with biotin [119].

Pereira-Silva et al. provided an innovative homotypic targeting by coating Soluplus^®^ micellar cores with purified cancer cell membranes extracted directly from pancreatic cancer cells. This biomimetic coating has been combined with a hydrophobic prodrug strategy to optimize the delivery of gemcitabine (highly hydrophilic with short biological half-life drug) [120].

Highly branched naturally derived polymers have been assembled as a macromolecular prodrug platform, where the structural degradation products themselves are active anti-cancer agents, creating a dual-action therapeutic synergy [121].

New architectures have also been explored, including dendrimers and cyclodextrin-based polymeric systems. Koti et al. engineered a core-tunable hyperbranched dendritic polymer whose molecular weight, branching density, and hydrophobic domain size can be precisely tuned via the monomer/diol-linker ratio, combining folate-guided targeting with near-infrared fluorescent tracking in a single theranostic platform [122].

The novelty within the literature of BPNPs lies in framing cyclodextrins not just as basic solubilizing enhancers but as dynamic, highly customizable macromolecular frameworks. By bridging polymer chemistry with stimuli-responsive behaviors, multidrug resistance inhibition, and theranostic multi-tasking, cyclodextrin-based polymeric systems represent one of the most promising frontiers for personalized, high-precision cancer therapy [123].

Consequently, emerging BPNPs are poised to transition towards multifunctional nanoplatforms, namely hybrid organic–inorganic systems (fourth-generation systems, as illustrated in Figure 1) and theranostic systems (fifth-generation systems, as illustrated in Figure 1). As noted above, these two categories overlap substantially in practice rather than representing discrete sequential stages. The subsequent sections of this review will delve into a detailed discussion of these novel systems, which are aimed at precision nanomedicine.

### 3.2. Lipid-Based Nanocarriers

Lipid-based NPs (LBNPs) have been engineered to address the critical bottlenecks of conventional cancer chemotherapy, such as poor drug aqueous solubility, rapid systemic clearance, and severe off-target toxicities, thanks to their high biocompatibility and capacity to passively accumulate within solid tumors through the enhanced permeability and retention (EPR) effect. The field of LBNPs has moved from “liposomes as drug carriers” to a broader toolbox of lipid-based nanocarriers that includes liposomes, solid lipid NPs, nanostructured lipid carriers, nanoemulsions, and lipid-polymer hybrids [124,125,126].

Among LBNPs, liposomes and polymeric/lipid micelles represent the most clinically mature and mechanistically well-characterized platforms, providing the conceptual foundation for the more advanced systems discussed below. Liposomes are spherical vesicles composed of one or more phospholipid bilayers enclosing an aqueous core, allowing encapsulation of both hydrophilic (aqueous core) and lipophilic (bilayer-embedded) drugs; PEGylation of the liposomal surface (“stealth” liposomes) reduces opsonization and MPS clearance, extending circulation half-life and promoting EPR-mediated tumor accumulation (Section 7.2.2). This design underlies several clinically approved anticancer liposomal formulations, including PEGylated liposomal doxorubicin (Doxil/CAELYX) and liposomal irinotecan (Onivyde), which reduce off-target toxicity (e.g., cardiotoxicity for doxorubicin) relative to the free drug while preserving efficacy [127,128,129]. Micelles, by contrast, are self-assembled nanostructures formed above the critical micelle concentration by amphiphilic block copolymers or lipids, with a hydrophobic core that solubilizes poorly water-soluble drugs and a hydrophilic corona (typically PEG) that confers colloidal stability and stealth behavior; their small size (typically 10–100 nm) and dynamic, non-covalent assembly mechanistically distinguish them from the bilayer architecture of liposomes. Polymeric micelles have reached clinical use for taxane delivery, exemplified by paclitaxel-loaded PEG-poly(D,L-lactide) micelles (Genexol-PM), which avoid the Cremophor-EL-related hypersensitivity reactions associated with conventional paclitaxel formulations (Section 1.1) [130]. Both platforms illustrate, at the clinical stage, the rationale for lipid-based nanomedicine introduced in Section 1.1: improved solubilization, reduced off-target toxicity, and EPR-mediated tumor accumulation.

As discussed for BPNPs, LBNPs have similarly evolved from passive carriers to engineered multifunctional platforms combining PEGylation with active targeting ligands (antibodies, aptamers, RGD peptides).

The frontier in lipid nanocarrier design is environmental responsiveness, achieved through lipids/polymers that undergo phase transitions upon intrinsic (acidic pH, GSH) or extrinsic (NIR, magnetic field, ultrasound) triggers. LBNPs increasingly co-encapsulate MDR-reversing chemotherapeutics with imaging agents in single theranostic platforms, an approach that remains largely at the preclinical stage. This should be distinguished from the clinical translation already achieved by simpler, single-function lipid nanocarriers without integrated imaging or MDR-reversal activity, such as PEGylated liposomal doxorubicin (Doxil/CAELYX) and the siRNA-lipid nanoparticle Onpattro, both of which are FDA-approved; no multifunctional theranostic LBNP combining MDR reversal with imaging in a single platform currently holds regulatory approval [125,128].

The rational design of phospholipid nanocarriers has expanded past simple liposomes to encompass ultra-deformable transferosomes, ethosomes, and invasomes that exploit membrane elasticity and fluid phase behavior for targeted tumor localization [131]. Phospholipid nanocarrier design further modulates bilayer packing, phase-transition temperature, surface charge, and curvature elasticity to tune loading and release. Edge-activator surfactants confer transferosomes with high deformability; ethanol-rich ethosomes fluidize both vesicle and stratum corneum lipids for transdermal melanoma therapy; invasomes exploit terpenes for deeper penetration; phytosomes and pharmacosomes covalently complex actives with phospholipids to maximize loading; and virosomes exploit viral fusogenic proteins to bypass endosomal entrapment [131].

In detail, recent breakthroughs in nanomedicine demonstrate the exceptional versatility of LBNPs hybrid platforms in overcoming biopharmaceutical limitations in cancer therapy. To address the challenge of formulating highly hydrophobic or “brick-dust” molecules without premature drug precipitation, Virzì et al. have utilized advanced nanostructured lipid carriers (NLCs) to encapsulate newly synthesized, highly potent inhibitors targeting alternative survival enzymes like Heme Oxygenase-1 (HO-1), demonstrating remarkable selectivity and tumor reduction in complex in ovo models of metastatic malignancies [132].

Concurrently, comparing structurally distinct systems has helped optimize natural therapeutic transport; for example, micellar zein vectors demonstrate superior stability and improved cellular internalizing kinetics over conventional PEGylated liposomes when delivering poorly soluble curcumin to glioblastoma spheroids [133]. In parallel, structural modifications to first-generation solid lipid NPs (SLNs) have resolved classic drug ejection issues. Integrating protoporphyrin IX into customized SLNs preserves the monomeric states of photosensitizers, enhancing active oxygen species production during multi-wavelength photodynamic therapy [134].

Combining natural building blocks with engineered materials has produced customizable interfaces: microfluidic-assembled, oleosin-coated lipid vesicles alter membrane fluidity for sustained release; replacing costly apolipoproteins with modified albumin creates dual-targeted lipoprotein-mimicking carriers; and encapsulating plant extracts in MPEG-b-PLA micelles improves water-dispersibility and induces apoptosis in cutaneous carcinomas [135,136,137].

Modern oncology platforms have also transitioned toward stimuli-responsive architectures and non-lamellar topographies. Reversible Addition–Fragmentation Chain Transfer (RAFT) polymerization has allowed the synthesis of advanced pH-responsive block copolymers containing tertiary amine segments; these polymers act as smart stabilizers for non-lamellar lyotropic liquid crystalline NPs, triggering controlled, internal mesophase shifts (from cubosomes to hexosomes) inside acidic tumor microenvironments [138]. This liquid crystalline architecture is highly effective for protecting complex payloads, as seen in monoolein-based bicontinuous cubic nanoplatforms that shield *Ulva rigida* marine macroalgae extract from premature degradation, facilitating localized photodynamic destruction of resistant pancreatic ductal adenocarcinomas [139].

Lipid architectures also drive immunotherapy: PEGylated phenyl-glycolipid nanocarriers targeted secondary lymphoid organs, preventing NKT-cell exhaustion and generating Th1-polarised anti-metastatic responses [140]. Carrier-free, self-assembled verteporfin NPs achieve theoretical 100% drug loading, combining light-dependent PDT with dark-condition oncogenic pathway disruption [141].

### 3.3. Core–Shell and Hybrid Organic–Inorganic Nanocapsules

Cancer nanomedicine is converging on modular core–shell and hybrid organic–inorganic architectures, combining an organic compartment for drug loading with an inorganic component for imaging, magnetism, phototherapy, or controlled release. Early examples, such as galactosylated polyaspartamide–gold-nanorod conjugates (Gal-PHEA-PEG-GNRs), coupled continuous chemotherapeutic release with NIR-triggered photothermal ablation and galactose-mediated hepatocarcinoma targeting, illustrating the transition from simple hybrids to integrated smart nanocapsules [142].

However, as highlighted across the collective spectrum of recent literature, contemporary nanomedicine has aggressively shifted this paradigm away from simple monofunctional encapsulation toward multifunctional, hierarchically ordered smart platforms where every individual structural component actively coordinates and executes distinct biological, therapeutic, or diagnostic subtasks [143,144].

Similarly, boronate-ester/polydopamine-coated amphiphilic polymers remain stable in circulation but respond to acidic pH and elevated ROS, enabling co-delivery of hydrophobic photosensitizers (e.g., zinc phthalocyanine) with combined PDT/PTT and MRI monitoring [145]. Wang et al. built a ZIF-8 (MOF)-core, SI-RAFT-grafted fluoropolymer-shell nanoplatform for 19F MRI-guided, switchable-hydrophilicity delivery of DOX [143].

Core-shell metal-organic frameworks (MOFs) are a significant area of active research. They are characterized by excellent colloidal stability and serve as an effective methodology for generating efficient drug delivery systems, multimodal imaging modalities, and synergistic therapeutic systems [146].

A hallmark of this structural maturation is the replacement of fluid lipid bilayers or unstable polymer chains with rigid, porous inorganic templates (MOFs, iron-based frameworks, calcium carbonate), which can protect sensitive payloads (DOX, paclitaxel, curcumin, SNX-2112) from premature degradation [147,148]. Burst release from these carriers is not intrinsically eliminated, however, and remains strongly dependent on pore architecture, the strength of drug–matrix interactions, gatekeeper or capping-layer stability, coating integrity, the loading method employed, the degree of dilution upon administration, and the physiological release medium. Indeed, directly comparable in vitro release studies on DOX-loaded MOF NPs have reported burst release of 29–42% of the total payload within the first 24 h [149], a magnitude comparable to, rather than systematically lower than, values reported for liposomal and micellar DOX formulations. Rigid inorganic scaffolds should therefore be regarded as offering the potential for improved control over burst release under favorable design conditions, rather than a general, inherent advantage over lipid- or polymer-based carriers. Shell design has similarly evolved toward multi-stimuli-responsive gating, with calcium carbonate particularly attractive for its abundance, biocompatibility and pH-dependent dissolution, stable at pH 7.4 but dissolving into non-toxic Ca^2+^/CO_3_^2−^ at tumor pH [150,151,152]. Dani et al. exploited this with PEG-coated, curcumin-loaded CaCO_3_ NPs disintegrating specifically at pH 5.0 [148].

Kordy et al. combined an NH_2_-MIL-101(Fe) MOF core (high surface area, high DOX loading) with a glutathione-targeted PAMAM dendrimer shell: the complex is stable at pH 7.4 but disassembles upon endo-lysosomal acidification (pH 5.0) via amine protonation, releasing DOX specifically in breast-cancer cells, while the dendrimer shell mimics PEGylated stealth behavior [147].

The premature leakage and non-specific distribution of self-assembled polymeric NPs remains a critical challenge in modern cancer nanomedicine. While polymeric systems are highly regarded for their ability to encapsulate poorly soluble chemotherapeutic drugs, conventional single-component micelles often suffer from structural instability upon dilution in the bloodstream. This instability leads to a “burst release” of the toxic cargo before it reaches the tumor site, causing systemic side effects and reducing therapeutic efficacy.

Liu et al. engineered an ATP/hyaluronidase dual-stimuli core-shell system: a methacrylate-hyaluronic-acid shell (CD40/CD44-targeting) is degraded by tumor-overexpressed hyaluronidase, exposing a polylysine-fluoro-carboxyphenylboronic-acid core that then disassembles in response to high intracellular ATP, replacing passive diffusion with active, biomarker-triggered release [153].

Superparamagnetic iron oxide (Fe_3_O_4_) NPs are a premier template for physical targeting and MRI contrast, though pristine cores are prone to oxidation and aggregation. Cengiz et al. stabilized them via grafting-from SI-RAFT polymerization, generating a biodegradable polymer-brush shell rich in functional groups for DOX loading and RGD-peptide targeting. Using a reversible Diels–Alder/retro-Diels–Alder linkage, this design merges magnetic cores with “smart” polymer brushes into a reliable platform for next-generation targeted therapy.

To resolve these surface limitations, Azari et al. coated a magnetic Fe_3_O_4_ core with a SiO_2_ shell functionalized with APTMS/NH_2_ and FA, combining passive EPR, folate-active targeting, and magnetic targeting to maximize local accumulation [154].

The severe systemic side effects of passive targeting and rapid clearance motivate active-targeting strategies combining ligand decoration with stimuli-responsive release, as discussed for individual nanocarrier classes above.

Metal-phenolic coordination networks offer a green alternative to synthetic surfactant coatings: a self-assembled Fe^3+^–EGCG shell around an oil-in-water nanoemulsion achieved high co-encapsulation of drugs with opposing polarities (hydrophobic paclitaxel in the lipid core, hydrophilic gemcitabine in the coordination shell) without requiring polymer synthesis [155].

To bridge the gap between these multi-component lab-scale platforms and clinical translation, manufacturing reproducibility and regulatory characterization remain the central challenges, as discussed in Section 10 [156].

It is evident that the 2022–2026 literature demonstrates a shift in focus from a singular triumph of a particular material to a more comprehensive approach that emphasizes the convergence of several technological advances. These advances include precise shell engineering, combined imaging and therapy within a unified platform, receptor targeting, and biomimetic multifunctional design.

The field is moving toward self-assembled hybrid systems that co-package a small-molecule drug with small interfering RNA (siRNA) so the two payloads can hit complementary pathways. The most visible advance is the move away from bulky, purely carrier-based formulations to address the “carrier dilemma.” While conventional nanomedicines rely heavily on exogenous delivery vehicles (such as synthetic polymers, silica templates, or liposomes) to transport therapeutic payloads, these inert carriers often introduce severe clinical complications, including low drug-loading capacity, systemic carrier-related toxicity, long-term tissue accumulation, and complex, non-reproducible multi-step synthetic pathways that hinder industrial scale-up.

A carrier-free strategy co-assembled a Survivin-targeting antisense oligonucleotide with the photosensitizer chlorin e6 via electrostatic and π–π interactions, yielding a near-100% active-payload nanoplatform for combined photodynamic and gene therapy [157].

Co-delivering siRNA and chemodrugs is hindered by their mismatched physicochemical properties. Xu et al. addressed this by directly self-assembling anti-P-gp siRNA with cationic amphiphilic chemodrugs into a carrier-free nanoparticle core, i.e., without any separate inert carrier phase, which was then camouflaged with an erythrocyte-membrane shell exploiting native CD47 to evade immune clearance. Because of this additional biomimetic membrane coating, the overall construct is more accurately described as a minimal-carrier, biomimetic nanoassembly rather than a fully carrier-free system; it was reported to prolong circulation more effectively than conventional PEGylation [158,159].

A related metal-coordination platform links the siRNA phosphate backbone to Fe^2+^ ions while co-encapsulating DOX; cRGD-functionalized lipid coating enables dual targeting of lung-cancer cells and tumor endothelium, combining gene silencing with DNA-intercalating chemotherapy while avoiding the toxicity of cationic polymer carriers [160].

Hybrid lipid-polymer systems remain the preferred engineering platform: microfluidic manufacturing enables co-loading of budesonide/eGFP-siRNA for high transfection at low dose, while other designs use a PLGA/cationic-lipid core encased in a lipid shell, partitioning hydrophobic drug in the core and condensing siRNA electrostatically at the core–shell interface [161].

In pulmonary delivery, another 2024 LPHN platform achieved about 99% siRNA encapsulation and about 50% gene silencing in human lung cancer cells, then retained activity after spray-drying for inhalation. The synthetic copolymer based on an α,β-poly(N-2-hydroxyethyl)-D,L-aspartamide (PHEA) backbone was strategically modified with PLGA to introduce controlled hydrophobic domains, spermine-like polyamine structures to provide dense cationic charges capable of effectively condensing the negatively charged siRNA, a fluorescent dye for tracking cellular uptake [162].

A 2024 pH-responsive liposomal platform co-delivered siRNA and docetaxel using a histidine-modified cRGD ligand: extracellular tumor pH (6.5) protonates histidine to enhance integrin binding, while endosomal pH (5.5) triggers ionizable-lipid charge switching and siRNA cytoplasmic release, achieving sequential, two-stage targeting and release [163].

The field converges on dual-hit systems for resistant breast, lung, and solid tumors, following a shared design logic (self-assembled/hybrid cores, one small-molecule drug plus one siRNA, intracellular-release trigger) where siRNA suppresses resistance/survival pathways, while the small molecule delivers cytotoxicity [164].

## 4. Stimuli-Responsive NPs and the Tumor Microenvironment

**Figure 2:**
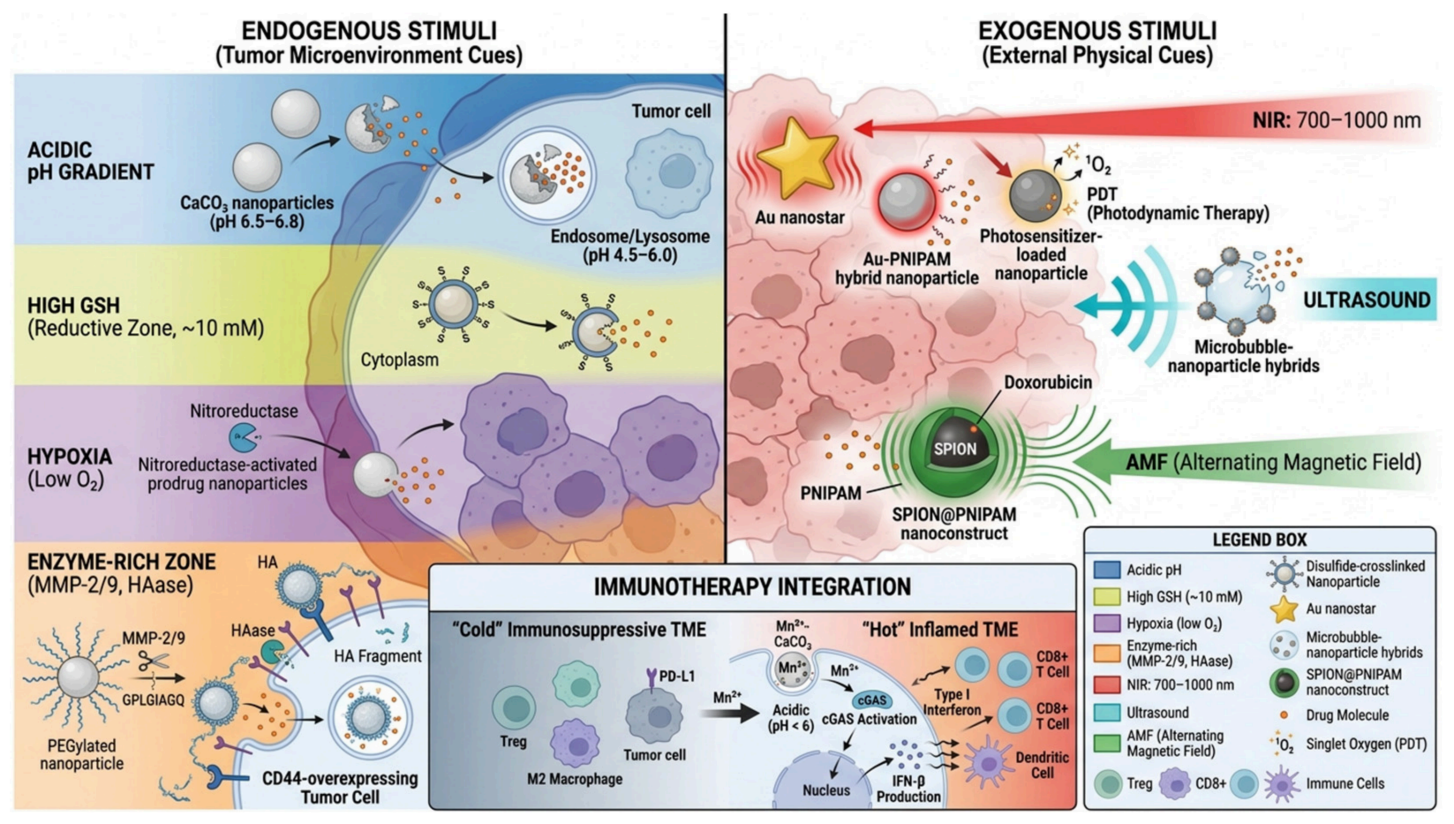
Endogenous (pH, GSH, hypoxia, enzyme) and exogenous (NIR, ultrasound, magnetic field) stimuli exploited for tumor-selective nanoparticle activation, including integration with cGAS–STING-mediated cancer immunotherapy. *During the preparation of this figure, the author(s) used FigureLabs to generate the graphical illustration of the endogenous and exogenous stimuli-responsive activation pathways, based on the authors’ original concept, structural layout, and scientific content. The authors have reviewed and edited the output and take full responsibility for the content of this figure*.

Exploiting TME abnormalities for site-selective release has transformed nanocarrier design: unlike EPR-dependent passive delivery, stimuli-responsive NPs are engineered to remain largely inert in circulation and to activate preferentially, rather than exclusively, within the tumor. Because the underlying pH, redox, hypoxic, and enzymatic cues also occur, to varying degrees, in inflamed, infected, healing, and even some healthy tissues, these systems remain susceptible to premature activation, threshold overlap, and intra- and inter-tumoral heterogeneity, as discussed in detail in Section 4.2. Endogenous triggers include acidic pH, elevated GSH, hypoxia, enzymes, and ROS [165,166]; exogenous triggers (NIR, ultrasound, heat, magnetic fields) add external control, increasingly coupled with immunotherapy [167] (Figure 2).

### 4.1. Features of the Tumor Microenvironment as Triggering Stimuli

#### 4.1.1. Acidic pH

Aerobic glycolysis causes lactic/carbonic acid accumulation, giving tumor extracellular pH 6.5–6.8 versus 7.4 in normal tissue, with endosomes (5.5–6.0) and lysosomes (4.5–5.0) more acidic still. These gradients are exploited via acid-labile bonds (hydrazone, ketal, acetal, Schiff base), proton-sponge polymers (imidazole/tertiary-amine-rich, causing osmotic swelling), and dissolution of acid-soluble inorganic carriers.

#### 4.1.2. Redox Imbalance: GSH and ROS

The GSH gradient between tumor cytoplasm (2–10 mM) and extracellular space (~2 μM) lets disulfide-crosslinked nanocarriers (PLGA, poly(amidoamine)) stay stable extracellularly but disassemble rapidly intracellularly, exploited for Pt(IV) prodrug delivery (~11% loading, ~80% release in 3 days) and GSH-depleting trisulfide prodrugs enhancing tumor-selective cytotoxicity [168,169,170].

ROS overproduction enables boronate-ester-linked nanocarriers that hydrolyze selectively under oxidative stress. Badparvar et al.’s dual pH/redox nanoplatform (PAA-b-PCL-S-S-PCL-b-PAA) shifted charge (−17.8 to −2.4 mV) and shrank (170 to 93 nm) in the TME, achieving 71.6% apoptosis in MDA-MB-231 cells versus 49.8% for free DOX [171].

#### 4.1.3. Hypoxia

Hypoxic tumor regions (pO_2_ < 5 mmHg) overexpress nitroreductases and azoreductases, enabling selective activation of nitro- or azo-bearing prodrug linkers, while HIF-1 α stabilization upregulates targetable receptors (GLUT-1, transferrin receptor). Hypoxia-activated TiO_2_-releasing platforms represent an emerging sub-category for aggressive, avascular tumors [172,173].

#### 4.1.4. Overexpressed Enzymes

Overexpressed TME proteases provide highly selective triggers. MMP-2/9 cleave peptide linkers (e.g., GPLGIAGQ, PVGLIG) that shed a nanocarrier’s protective shell [174,175]; cathepsin B cleaves Val-Cit/Phe-Lys bonds and is widely exploited in antibody–drug conjugates and peptide-gated NPs [176,177,178]; hyaluronidase-responsive hyaluronic-acid shells enable CD44-targeted, on-demand release [179,180]. These enzymes are overexpressed, rather than exclusively present, in tumor tissue: MMPs, cathepsins, and hyaluronidases remain constitutively active at lower levels in healthy tissue and can be further upregulated in inflammatory and wound-healing contexts, so enzyme-responsive designs achieve preferential rather than absolute tumor selectivity and remain susceptible to premature activation and off-target release under these conditions (Section 4.2).

### 4.2. Endogenous Stimuli-Responsive Nanocarrier Platforms

Endogenous stimuli-responsive nanocarriers exploit tumor-associated cues (acidic pH, elevated glutathione, overexpressed enzymes such as MMPs, cathepsins, and hyaluronidases) to trigger site-selective release. However, these stimuli are not tumor-exclusive—similar conditions occur in inflamed, infected, or healing tissues, and the target enzymes remain constitutively active, at lower levels, in healthy tissue. This exposes such systems to premature activation, off-target release, and variable performance due to intra- and inter-tumoral heterogeneity, limiting their clinical translatability compared with other nanocarrier strategies. Zhou et al. (2022) classified these as charge-switchable, size-transformable, or bond-cleavable designs, each with distinct TME-triggered release kinetics [166].

Among pH-responsive platforms, CaCO_3_-based nanocarriers combine drug delivery with acid-neutralizing activity, dissolving into non-toxic Ca^2+^/CO_3_^2−^ in the acidic TME while remaining stable at physiological pH [181]. Layer-by-layer PBAE/hyaluronic-acid shells add endosomal-pH-triggered swelling with CD44-mediated targeting [182], while hydrazone-linked, charge-reversal nanovehicles switch from neutral (pH 7.4, long circulation) to positively charged and size-reduced at tumor/endosomal pH, enabling sequential matrix penetration and intracellular release [183].

Redox-responsive systems exploit the same GSH gradient as pH designs: disulfide-crosslinked NPs remain stable physiologically but disassemble completely upon endocytosis, with concomitant GSH depletion sensitizing tumor cells to oxidative stress [184].

Enzyme-responsive designs exploit overexpressed TME proteases for tumor-selective activation. MMP-2-cleavable GPLGIAGQ linkers shed the protective PEG corona at the tumor site, enhancing internalization and spheroid penetration, and have been extended to MMP-cleavable antibody–drug conjugates [185,186]. Hyaluronidase-responsive systems add a second trigger, with surface HYAL2 and lysosomal HYAL1 sequentially degrading HA shells for CD44-targeted intracellular release [187].

Advanced platforms combine multiple responsive modalities: dual HAase/pH-responsive HA-coated poly(beta-amino ester) micelles achieve simultaneous CD44-targeted endocytosis and pH-triggered release, selectively eliminating CD44-overexpressing cancer stem cells [188]; dual pH/redox nanocarriers likewise show superior in vivo tumor inhibition versus single-stimulus controls [166].

### 4.3. Exogenous Stimuli-Responsive Systems

Exogenous stimuli give clinicians spatial/temporal control via external devices. NIR light (700–1000 nm, 5–10 mm penetration) is preferred for accessible tumors, minimizing phototoxicity [189,190]; gold nanorods/nanostars absorb NIR with extraordinary efficiency, generating hyperthermia that destabilizes thermoresponsive Au-PNIPAM coatings [191,192,193,194,195].

Photosensitizer-loaded NPs (chlorin e6, zinc phthalocyanine, novel organometallic agents) generate singlet oxygen upon laser irradiation for PDT with bystander cell killing [196,197,198,199,200].

Photo-responsive Pt complexes (Pt-curcumin, azobenzene-Pt) in PLGA NPs combine Pt-mediated DNA crosslinking with photoactivatable cytotoxicity, a dual mechanism reducing acquired resistance [127,200,201].

Focused ultrasound (FUS) offers deep tissue penetration combined with good spatial precision, using acoustic cavitation to disrupt nanoparticle shells and enhance uptake (sonoporation); microbubble-nanoparticle hybrids add real-time ultrasonographic monitoring. Alternating magnetic fields (100–500 kHz) achieve comparable or greater penetration depth but with lower spatial resolution, triggering magnetothermal release from thermoresponsive coatings; SPION@PNIPAM constructs achieve synergistic chemo-thermal tumor suppression without added systemic toxicity, though clinical use of both modalities remains constrained by equipment requirements and safety limits on energy deposition [202,203,204,205].

### 4.4. TME-Responsive NPs in Cancer Immunotherapy

Integrating smart nanocarriers with immunotherapy is arguably the most impactful recent advance: the immunosuppressive TME (Tregs, MDSCs, M2 TAMs, TGF-beta/IL-10/PD-L1) limits checkpoint blockade efficacy to a minority of patients, motivating nanoparticle strategies that convert immunologically “cold” tumors to “hot” by re-modeling the TME while delivering immunogenic payloads.

STING agonism is among the most powerful immunotherapeutic targets: cytosolic DNA sensing activates cGAS-STING signaling, driving type-I interferon production, dendritic-cell maturation, and CD8+ T-cell priming, and potentiating PD-1/PD-L1 blockade [206,207]. Because free STING agonists (cGAMP, DMXAA, diABZI) cause severe immune-related toxicity, nanocarriers (lipid, polymeric, MOF, mesoporous silica, exosomal, hydrogel) are used to achieve 10- to 100-fold higher intratumoral concentration with reduced systemic exposure.

Mn^2+^-doped CaCO_3_ NPs achieve simultaneous pH-triggered drug release and STING activation: TME dissolution releases both Mn^2+^ (catalysing cGAMP production) and the chemotherapeutic payload, creating a two-in-one immune activator/drug carrier. Similarly, co-delivery of ICD-inducing chemotherapeutics with TLR agonists or IDO inhibitors elicits systemic antitumor immunity controlling distal tumors in preclinical breast and colorectal cancer models [208,209,210,211,212,213].

The overarching lesson is that effective next-generation nanomedicines will act not only as drug carriers but as active remodelers of the tumor immune landscape.

## 5. Biomolecules and Natural Compounds as Therapeutic Agents

The diverse biomolecule- and natural-compound-based strategies discussed in this section—spanning phytochemicals, polysaccharides, nucleic acids, and targeting peptides—share a common translational goal: overcoming the pharmacokinetic and delivery limitations of free bioactive compounds through rationally engineered nanocarriers. Table 2 summarizes the main compound classes covered in this section, together with their representative delivery systems, mechanisms of action, key experimental findings, and, where applicable, stimuli-responsive, theranostic, or AI-guided design features. This comparative overview highlights how, across otherwise heterogeneous biomolecule classes, convergent engineering principles—tumor-microenvironment-responsive release, multifunctional imaging–therapy integration, and computationally assisted carrier optimization—are increasingly being adopted to improve targeting specificity and therapeutic efficacy in cancer nanomedicine.

### 5.1. Polyphenols, Carotenoids, and Other Phytochemicals

Phytochemicals modulate multiple cancer hallmarks in preclinical models, but poor oral bioavailability, rapid metabolism, and low solubility limit their clinical use; pharmacokinetics remains the key bottleneck, motivating nano-delivery systems to bridge this gap [214]. As summarized in Table 2, the strength of supporting evidence differs markedly across compound classes, ranging from cell-free physicochemical or antioxidant assays, through cellular uptake and in vitro cytotoxicity, to animal efficacy and, occasionally, early pharmacokinetic or human data; this evidence level is indicated for each compound below and should be borne in mind when interpreting the comparative statements that follow.

Polyphenols—flavonoids (e.g., quercetin), stilbenes (e.g., resveratrol), phenolic acids, and curcuminoids (e.g., curcumin)—dominate the field, with polymeric and lipid NPs, nanoemulsions, phytosomes, and hydrogel-based formulations consistently improving solubility and cellular uptake relative to free compounds, and, in the compound classes for which such data exist (Table 2), also improving in vitro cytotoxicity or in vivo antitumor activity [215].

Curcumin, the most extensively studied curcuminoid, has broad pharmacological activity but severely limited bioavailability, with plasma levels of only ~2.30 µg/mL after a 10 g oral dose due to hydrophobicity and rapid hepatic metabolism [216,217,218]. Nanoencapsulation in liposomes, SLNs, NLCs, micelles, cyclodextrin complexes, dendrimers, or BPNPs restores solubility and stability and enables active targeting via FA, transferrin, antibody, or peptide functionalization [219,220]. A 2022 meta-analysis of 18 rodent studies confirmed substantially reduced tumor growth and weight after encapsulation compared with free curcumin [221], and layered double hydroxide nanocurcumin has proven an effective apoptotic platform against lung cancer in vivo [222].

Lycopene (LYC), a potent dietary antioxidant, is limited clinically by extreme hydrophobicity and oxidative instability [223,224,225]. Nanoemulsions, SEDDS, liposomes, and polymeric NPs improve its solubility and cellular delivery; LYC -loaded amphiphilic peptide nanomicelles increased intracellular accumulation in intestinal epithelial cells ~2.2-fold relative to free LYC via ER–Golgi–lysosomal trafficking [226].

Surfactant selection critically modulates NPs–cell interactions: Tween 20-stabilized PLGA-LYC NPs showed smaller size and greater internalization into skin melanocyte spheroids than PVA-stabilized counterparts, whereas PVA improved long-term LYC retention [102].

Thymol (THY), a monoterpenoid phenol with antioxidant and anticancer activity, is limited by volatility and poor aqueous solubility [227]. Thymol-loaded solid lipid NPs developed for colorectal cancer achieved sustained release and significantly higher cytotoxicity against HT-29 cells than free thymol [228], while metallic nanoplatforms co-loading thymol with imaging or photothermal function point toward multifunctional theranostic applications [229,230].

Flavonoids and anthocyanins share broad antioxidant and anticancer mechanisms but similarly require nanocarrier-based delivery to overcome poor bioavailability and rapid metabolism [231,232,233,234]. Nanoencapsulation consistently improves radical-scavenging capacity relative to free compounds in cell-free assays: thermoresponsive nanoparticle-encapsulated lycopene halved the IC50 in a DPPH radical-scavenging assay compared with free LYC, and curcumin-loaded casein complexes increased compound half-life roughly 39-fold, from 8.8 to 340 min. As indicated in Table 2, these DPPH-based findings reflect antioxidant chemistry rather than anticancer efficacy and are not, by themselves, evidence of antitumor activity; the physicochemical gains described here are attributed to enhanced bioavailability (up to 47-fold for fisetin liposomes) [231,232], sustained release, and improved intracellular uptake through endocytosis.

Collectively, nanoencapsulation converts poorly bioavailable phytochemicals into multifunctional—and, increasingly, stimuli-responsive or theranostic—delivery platforms; however, as summarized in Table 2, the strength of supporting evidence varies substantially across compound classes, from cell-free physicochemical or antioxidant data and in vitro cytotoxicity to animal efficacy and, rarely, human evidence, and only the latter categories support claims of anticancer efficacy. Scale-up reproducibility and the mechanistic characterization of oral absorption remain additional key translational barriers [102].

### 5.2. Natural Polysaccharides: Inherent Bioactivity and Carrier Properties

Natural polysaccharides from marine, plant, and bacterial sources are increasingly recognized as bioactive materials rather than inert excipients. Their functional groups (hydroxyl, carboxyl, amino, sulfate), biodegradability, low immunogenicity, and tunable chemistry support NPs, nanogels, hydrogels, and hybrid nanocomposites with controlled drug release for next-generation cancer therapy [235,236,237,238].

Among marine polysaccharides, chitosan’s cationic, mucoadhesive character and pH-sensitive protonation support acidic-tumor-microenvironment (TME)-selective release, as shown by chitosan/alginate NPs loaded with amygdalin and gefitinib-alginate-chitosan carriers that markedly reduced lung-cancer IC50 versus free drug [239,240]. Carrageenan, though less bioactive itself, is a versatile stimuli-responsive carrier, exemplified by κ-carrageenan/chitosan hydrogels for pH-responsive sunitinib release and iota-carrageenan/β-cyclodextrin systems for methotrexate delivery [239,241].

Fucoidan combines carrier functionality with intrinsic antiangiogenic (VEGF/VEGFR suppression), immunomodulatory (STING–TBK1–IRF3 activation, PD-L1 downregulation), and antioxidant activity, and has been incorporated into P-selectin-targeted nanocarriers capable of crossing the blood–brain barrier via caveolin-1-mediated transcytosis [241,242]. Fucoidan NPs improve tumor accumulation and efficacy of doxorubicin, paclitaxel, and cisplatin while reducing systemic toxicity [243].

Hyaluronic acid (HA) is the most extensively exploited targeting polysaccharide, owing to its affinity for CD44 receptors overexpressed in many tumors; HA-functionalized NPs have improved tumor-specific delivery of doxorubicin, icaritin, and let-7b miRNA [244,245]. Several stimuli-responsive HA systems have been engineered for on-demand release: cystamine-modified HA responds to elevated intracellular glutathione (redox-responsive), 2,3-dimethylmaleic anhydride-functionalized HA degrades selectively under acidic tumor/endosomal pH, and azobenzene-based HA nanocapsules enable photo-responsive release [239,244,246]. Other polysaccharides add receptor-specific targeting—galactose/glucose polymers for hepatic ASGPR, alginate nanomicelles for macrophage mannose receptors, and pectin RG-I fractions for Galectin-3 in hepatocellular carcinoma [245].

Bacterial exopolysaccharides (dextran, pullulan, gellan gum, bacterial cellulose, levan, curdlan) offer improved manufacturing scalability and structural control through fermentation optimization, and several also act as intrinsic immunomodulators, supporting integration into multifunctional theranostic platforms that combine drug delivery, immune regulation, and imaging [238].

Overall, polysaccharide-coated NPs combine active targeting, receptor-mediated internalization, and stimuli-responsive release for precision cancer therapy. Clinical translation remains constrained by structural heterogeneity and manufacturing reproducibility, motivating future work on precision-engineered, AI-assisted polysaccharide design integrated with theranostic function.

### 5.3. Nucleic Acid-Based Therapeutics

Nucleic acid-based therapeutics act directly at the genetic level—through gene silencing, restoration of tumor-suppressor function, or immune reprogramming—via siRNA, miRNA, antisense oligonucleotides (ASOs), lncRNA-targeting strategies, and mRNA platforms, offering specificity unattainable with conventional protein-inhibiting drugs. Clinical translation is constrained by nuclease degradation, poor membrane permeability, and endosomal entrapment; delivery systems—LNPs, polymeric NPs, dendrimers, exosomes, inorganic carriers—protect nucleic acid cargo, and ionizable lipids specifically acquire positive charge in the acidic endosome to destabilize the membrane and trigger cytoplasmic release [246,247,248].

#### 5.3.1. Long Non-Coding RNAs (lncRNAs): Emerging Regulators in Cancer Biology

Long non-coding RNAs (lncRNAs) are a major class of regulatory RNA molecules that, despite lacking protein-coding capacity, play essential roles in gene regulation and cellular homeostasis. Once considered transcriptional noise, lncRNAs are now recognized as functional regulators involved in chromatin remodeling, transcriptional control, RNA stability, signaling pathways, and protein–RNA interactions. Their dysregulation is strongly associated with cancer initiation, progression, metastasis, and therapeutic resistance.

Oncogenic lncRNAs such as HOTTIP, CRNDE, LINK-A, HULC, and MALAT1 promote proliferation and survival pathways, whereas tumor-suppressive lncRNAs (SATB2-AS1, lincRNA-p21) inhibit growth or enhance treatment sensitivity; MALAT1 also drives multidrug resistance by upregulating efflux transporters. The stability of lncRNAs in blood, plasma, and urine supports their use as diagnostic and prognostic biomarkers, relevant to theranostic strategies that pair molecular profiling with targeted nanoparticle delivery [249].

#### 5.3.2. Antisense Oligonucleotides (ASOs) and mRNA Therapeutics: Lessons from LNP Delivery Systems

ASOs suppress gene expression through RNase H1-mediated degradation or splicing modulation (e.g., nusinersen for spinal muscular atrophy), while mRNA therapeutics deliver translatable genetic information into the cytoplasm; ionizable lipids that remain neutral in circulation but protonate in acidic endosomes have been the key LNP innovation enabling both modalities [246,247].

Clinical validation is well established—patisiran established systemic LNP-mediated RNA therapy, and COVID-19 mRNA vaccines confirmed in vivo mRNA delivery—though the MRX34 trial failure underscored the need to control innate immune activation. Remaining challenges include anti-PEG antibody formation from PEGylated LNPs and predominant hepatic accumulation via ApoE/LDLR uptake; selective organ targeting (SORT) strategies, AI-assisted design of ionizable lipids and RNA sequences, and microfluidic manufacturing are improving stability, encapsulation efficiency, and reproducibility for next-generation precision-oncology applications [246,247,248].

#### 5.3.3. Nanoparticle-Mediated Co-Delivery of Small Molecules and Nucleic Acids: Synergistic Approaches

Nanoparticle-mediated co-delivery of chemotherapeutics with siRNA, miRNA, ASOs, or mRNA combines direct cytotoxicity with gene regulation to overcome monotherapy limitations, enhancing apoptosis and reversing multidrug resistance. LNPs, polymeric NPs, liposomes, and hybrid nanocarriers protect both cargo types while enabling synchronized, stimuli-responsive release triggered by acidic pH, reductive conditions, or enzymatic activity in the tumor microenvironment, with ionizable lipids facilitating endosomal escape.

Preclinical co-delivery of doxorubicin, cisplatin, or paclitaxel with tumor-suppressive miRNAs/siRNAs targeting oncogenes or resistance pathways has improved efficacy while reducing systemic toxicity. Clinical translation is still limited by co-encapsulation of cargos with different physicochemical properties and large-scale manufacturing; current efforts focus on stimuli-responsive NPs, SORT-LNPs, and AI-assisted design strategies to integrate chemotherapy, gene regulation, and immunomodulation within single multifunctional nanoplatforms for precision oncology.

### 5.4. Peptides and Protein Carriers

#### 5.4.1. Human Serum Albumin as a Biocompatible Coating Agent and Targeting Vector

Human serum albumin (HSA), the most abundant plasma protein (~66.4 kDa), is a biocompatible, low-immunogenicity carrier that can exploit several endogenous transport pathways described for albumin-based systems, including gp60 (albondin)-mediated transcytosis, FcRn-mediated recycling, and SPARC-mediated retention in tumor stroma. However, the relative contribution of each pathway, and indeed the extent of tumor accumulation itself, varies considerably with tumor type, SPARC expression level, nanoparticle size and surface chemistry, and the specific albumin-based formulation used, and these mechanisms should not be assumed to operate uniformly across all albumin-coated nanocarriers. Similarly, while proliferating tumor cells can take up albumin as a nutrient source, and this has been proposed to allow HSA-based carriers to partially circumvent P-glycoprotein-mediated efflux in some models, this effect is context-dependent and has not been established as a general property of albumin-coated NPs. Overall, HSA offers a biocompatible platform with the potential for tumor-selective accumulation through multiple, partially overlapping mechanisms, though the dominant pathway and its clinical relevance likely differ across tumor types and formulations [250].

#### 5.4.2. Cell-Penetrating Peptides (CPPs): Enhancing Intracellular Delivery

Cell-penetrating peptides (CPPs) transport nucleic acids, proteins, and small molecules across membranes via cationic residues (arginine, lysine) that drive electrostatic interaction with cell membranes, entering cells through direct penetration or macropinocytosis-dominated endocytosis. Endosomal trapping remains a key limitation, prompting tumor-microenvironment-responsive designs: pH-low insertion peptides insert selectively under acidic tumor conditions, activatable CPPs are cleaved by tumor-associated matrix metalloproteinases, and hypoxia-responsive CPPs exploit the low oxygen tension of solid tumors to achieve selective intracellular delivery.

CPP conjugates with doxorubicin, methotrexate, or paclitaxel increase antitumor activity and reduce off-target toxicity, and CPP-functionalized nanocarriers can cross the blood–brain barrier, supporting targeted delivery to brain tumors [251].

#### 5.4.3. Tumor-Targeting Peptides: RGD, iRGD, and cRGD Conjugation to NP Surfaces

Most integrin-targeting peptides in current use, including RGD, cRGD, and iRGD, were identified through conventional rational peptide engineering and phage-display-based screening rather than through AI-driven design [252,253]; these approaches remain the primary discovery route for clinically advanced constructs to date. The most clinically mature example, certepetide (also known as CEND-1 or LSTA1), is an iRGD-derived cyclic peptide developed via this conventional route, exploiting the CendR/neuropilin-1 tumor-penetration mechanism originally characterized by Sugahara et al. and Teesalu et al. [253,254]. It is currently in multiple clinical trials for solid tumors, including a phase 2b placebo-controlled study in metastatic pancreatic ductal adenocarcinoma (ASCEND, NCT05042128), a phase 1b/2a study combined with neoadjuvant FOLFIRINOX-based regimens in pancreatic, colon, and appendiceal cancers (CENDIFOX, NCT05121038) [255], and separate trials in locally advanced/unresectable PDAC and first-line glioblastoma multiforme; preliminary reports describe favorable safety and encouraging trends in progression-free survival and objective response, though results remain preliminary and indication-specific and should not be generalized across tumor types. Genuinely AI-assisted approaches—using generative or predictive models to design novel binding peptides de novo—represent a distinct and still-emerging strategy, exemplified by recent deep-learning frameworks for target-specific peptide design [256], and remain largely at the preclinical stage; they should not be conflated with the discovery history of currently clinically tested RGD-family peptides. Taken together, RGD-, cRGD-, and iRGD-functionalized NPs illustrate how receptor-specific targeting and enhanced tumor penetration can improve therapeutic efficacy while limiting systemic toxicity, for example through improved lung-cancer drug accumulation with cRGD-modified albumin NPs [257], enhanced photothermal ablation in triple-negative breast cancer models [258], increased T-cell infiltration and immunotherapy efficacy in gastric cancer [259], and improved antisense-oligonucleotide delivery to prostate cancer bone metastases [260], with computationally guided peptide design representing a promising but separate future direction.

## 6. Bioinspired and Biomimetic Delivery Systems

**Figure 3:**
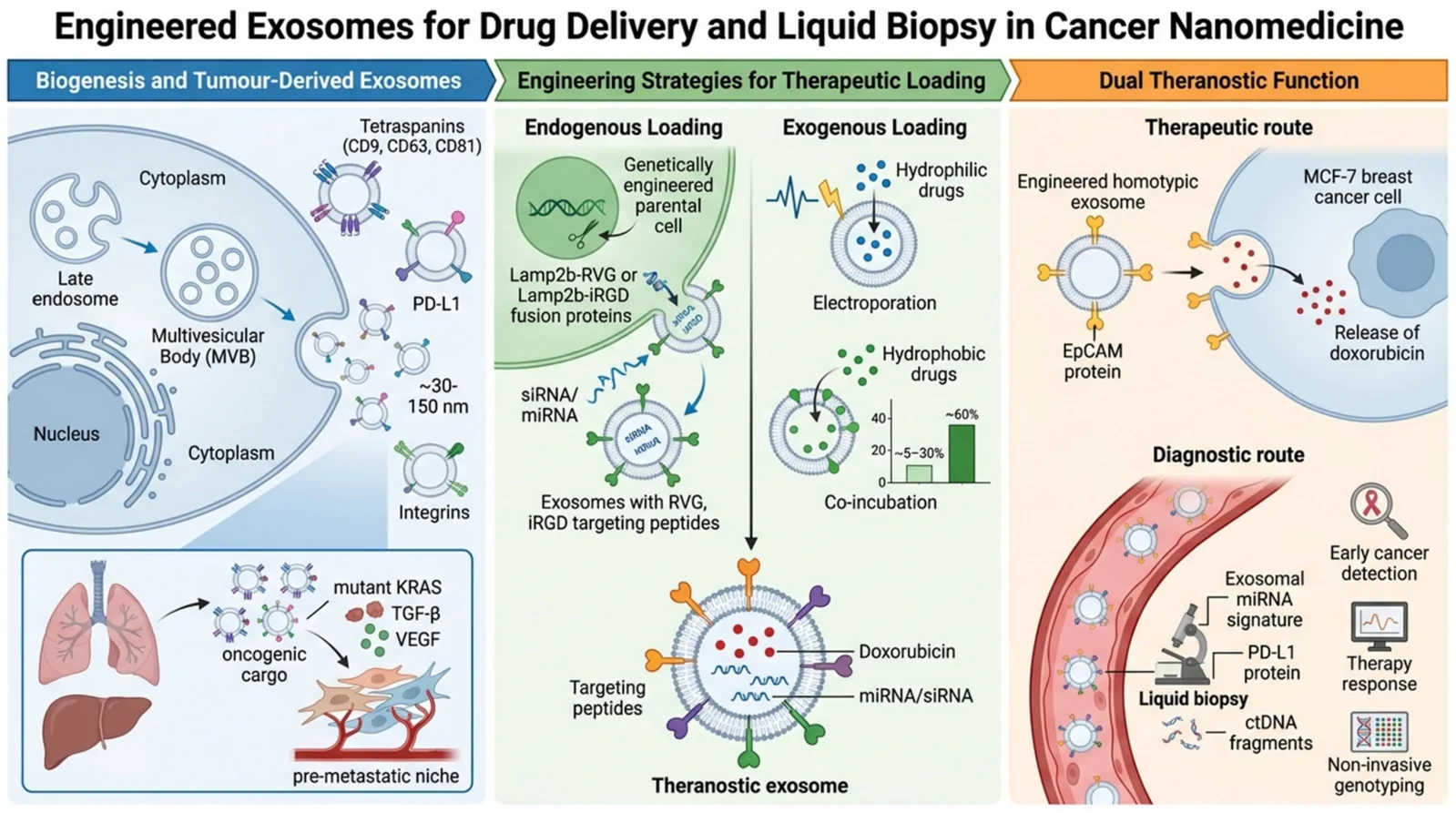
Exosome biogenesis and tumor-derived cargo, engineering strategies for endogenous and exogenous therapeutic loading, and dual theranostic application combining homotypic tumor-targeted drug delivery with liquid biopsy-based diagnostics. *During the preparation of this figure, the author(s) used FigureLabs to generate the graphical illustration of exosome biogenesis, engineering strategies and theranostic application, based on the authors’ original concept, structural layout, and scientific content. The authors have reviewed and edited the output and take full responsibility for the content of this figure*.

Synthetic delivery systems face persistent mononuclear phagocyte system (MPS) clearance, protein-corona-mediated loss of targeting function, and poor tumor-stroma penetration. Biomimetic nanomedicine addresses these barriers by emulating natural transport systems (extracellular vesicles, lipoproteins, viruses), either engineering cell-secreted vesicles or cloaking synthetic cores with native membranes, combining synthetic scalability with biological immune evasion and targeting [261] (Figure 3).

### 6.1. Exosomes and Extracellular Vesicles as Drug Delivery Vehicles

#### 6.1.1. Biogenesis and Natural Functions of Exosomes

Exosomes are 30–150 nm endosome-derived vesicles secreted by virtually all cells and upregulated by hypoxia/stress/oncogenic signaling; their surface proteome (CD9/63/81, HSP70/90, MHC-I/II, integrins) determines organ tropism. Tumor-derived exosomes carry oncogenic cargo (mutant KRAS, EGFRvIII), immunosuppressive molecules (PD-L1, TGF-beta), VEGF, and P-glycoprotein, contributing to pre-metastatic niche formation and immune evasion [262].

These same properties can be repurposed therapeutically: engineered exosomes from immune cells, MSCs, or tumor cells act as Trojan-horse vehicles delivering cytotoxic drugs, adjuvants, or nucleic acids with high specificity and low immunogenicity. A 2025 review by Liu et al. catalogued engineering methods enabling subcellular-organelle-targeted delivery (lysosomes, mitochondria, nuclei, ER) beyond simple cyto-plasmic delivery [263].

#### 6.1.2. Strategies for Therapeutic Loading of Exosomes

Exosome cargo loading follows two strategies: endogenous (engineering the parental cell during biogenesis) and exogenous (permeabilizing pre-isolated exosomes), each with different trade-offs in efficiency, cargo integrity, and scalability.

Endogenous loading is the gold standard for nucleic acids (siRNA, miRNA, mRNA), exploiting the cell’s RISC/RNA-sorting machinery; Lamp2b-fusion targeting peptides (RVG for BBB crossing, iRGD for tumor penetration, GE11 for EGFR targeting) enable scalable surface modification without compromising vesicle integrity, though genetic manipulation of the producer cell complicates Good Manufacturing Practice (GMP) manufacturing [264,265,266,267].

Exosome loading methods include co-incubation (hydrophobic drugs), electroporation (hydrophilic drugs/oligonucleotides), saponin-assisted permeabilization, freeze-thaw, and sonication, with a stratified approach combining electroporation and co-incubation to maximize efficiency [263]. Loading method, cargo chemistry, and exosome source all affect encapsulation efficiency: for small hydrophobic drugs such as doxorubicin, co-incubation typically achieves loading efficiencies in the range of 5–30%, whereas optimized electroporation protocols have reported up to ~60% for comparable cargoes, with efficiency generally defined as the proportion of total input drug recovered inside purified vesicles after removal of free drug, quantified by UV-Vis/fluorescence spectroscopy or HPLC [263,268]. These figures are illustrative ranges drawn from the cited reports rather than fixed or universal values and can vary substantially with vesicle source, cargo type, and the specific quantification protocol used; saponin-assisted loading gives the highest DOX content among the methods compared here but reduces membrane integrity [268].

#### 6.1.3. Tumor-Homing Exosomes and Liquid Biopsy Applications

Tumor-derived exosomes profiling is an active liquid-biopsy platform: exosomal miRNA signatures are candidate biomarkers in pancreatic, lung, and breast cancer [269,270]; exosomal PD-L1 tracks clinical response to anti-PD-1 therapy in melanoma [271]; and exosomal mutant-KRAS DNA enables pancreatic-tumor genotyping with higher detection rates than cell-free DNA [272]. Combining these diagnostic capacities with drug loading in a single “theranostic exosome” is an increasingly tractable goal [262].

Homotypic exosomes (derived from the same cancer cell line as the target) show enhanced tumor-homing: DOX-loaded homotypic EVs reduced parental-cell viability more effectively than free drug in breast cancer models [273], and (sinoporphyrin sodium)-loaded homotypic exosomes showed 1.8-2.4-fold higher uptake in matched versus heterologous cells [274], supporting self-targeting exosomal delivery design.

#### 6.1.4. Regulatory Challenges and Scalability

Clinical translation continues to face production bottlenecks: ultracentrifugation yields poor reproducibility, while higher-purity alternatives like size exclusion chromatography, asymmetrical-flow field-flow fractionation, and immunoaffinity isolation still require validation. Although the 2024 MISEV2023 guidelines provide essential reporting and characterization recommendations, they do not by themselves standardize manufacturing processes or establish regulatory acceptance [275]. Consequently, substantial variability remains, compounded by unsettled regulatory frameworks where the FDA classifies these products as biologics, and the EMA relies strictly on a reflection paper.

### 6.2. Cell Membrane-Coated NPs

A more scalable biomimetic strategy coats synthetic cores (PLGA, iron oxide, gold, MSN, lipid) with membranes extracted by hypotonic lysis/extrusion. This process transfers a substantial, but incomplete, subset of the source membrane’s proteome and lipidome: lysis, purification, and extrusion cause partial loss of membrane components, protein reorientation (including inside-out insertion), compositional shifts relative to the native membrane, and reduced or altered activity of some surface proteins. The resulting coating therefore confers immune evasion, targeting, and receptor-mediated uptake only to the extent that these functionally relevant components survive processing [276,277].

Erythrocyte membrane coating displays CD47/glycophorin A, engaging macrophage SIRPα for a “don’t eat me” signal and reducing complement opsonization [278,279]; platelet membrane coating exploits natural adhesion to vascular injury sites and circulating tumor cells via P-selectin/integrin αIIbβ3, addressing CTC-mediated metastasis [280,281].

Macrophage-membrane coating confers chemokine-receptor-driven (CCR2, CXCR4) homing into hypoxic tumor cores otherwise inaccessible to passively targeted particles, achieving threefold higher intratumoral accumulation than PEGylated controls in vivo [282,283,284]. Cancer-cell-membrane-cloaked NPs exploit homotypic adhesion (e.g., Ep-CAM-EpCAM) for self-targeting, improving uptake in matched tumor spheroids [285].

Hybrid membrane NPs fuse two or more cell-type membranes: RBC-platelet hybrid-coated NPs co-delivering DOX and anti-PD-1 achieved extended circulation (RBC) plus CTC targeting (platelet) for potent chemo-immunotherapy in murine models.

## 7. Bridging the Gap: Advanced 3D In Vitro Platforms and in Vivo Models in NPs Development

### 7.1. Advanced 3D in Vitro Models

#### 7.1.1. Tumor Spheroids: Formation, Characterization, Drug Penetration Studies

Multicellular tumor spheroids (MCTSs) recreate the density and metabolic gradients of solid tumors in vivo, generated via scaffold-free hanging-drop, ultra-low-attachment, or forced-aggregation techniques [286,287]. Spheroid growth and viability are monitored by live-cell imaging and fluorescent probes (Calcein-AM for viable cells, PI/ethidium homodimer for the necrotic core, hypoxia-sensitive dyes for the oxygen gradient) [288], while fluorophore-labeled nanoformulations allow confocal or light-sheet tracking of drug penetration kinetics.

These studies consistently demonstrate the robust barrier role exerted by the 3D architecture: while low-molecular-weight free chemotherapeutics penetrate relatively quickly but non-specifically into the MCTS, conventional NPs (>100 nm) remain confined to the outer proliferative shell due to the high density of the ECM and elevated interstitial hydrostatic pressure. This provides a clear explanation for why many vectors fail to reach the cancer stem cells nested within the hypoxic core (see Section 7.2.2 for the distinction between whole-tumor accumulation, as measured by bulk biodistribution assays, and true intratumoral distribution).

#### 7.1.2. Organoids Derived from Patient Biopsies: Personalized Drug Testing

Organoids represent a further evolutionary step: unlike immortalized spheroid lines, they are derived from patient-biopsy stem cells cultured in biomimetic hydrogels (e.g., Matrigel) with defined growth-factor cocktails (Wnt, R-spondin, EGF), preserving the histopathology, heterogeneity, and mutational profile of the original tumor [286,287,288].

Patient-derived organoids (PDOs) enable preclinical screening of multiple nanoformulations on a genetically matched model, identifying resistance mechanisms and validating targeted NPs (e.g., against specific neoantigens) prior to clinical administration, shortening regimen-selection time while limiting unnecessary patient exposure to ineffective treatments.

#### 7.1.3. Human Skin Spheroids: Evaluation of Nanoparticle Uptake for Dermal and Topical Applications

Beyond oncological modeling, 3D systems find significant application in the study of dermal toxicology and the development of topical formulations. Human skin spheroids and reconstructed human epidermis (RHE) models are generated by co-culturing immortalized human keratinocytes (e.g., the HaCaT line) and dermal fibroblasts within collagen matrices. These constructs are subsequently exposed to an air–liquid interface to stimulate cellular differentiation and the formation of the stratum corneum [289].

These 3D cutaneous models are employed to evaluate the safety and transdermal penetration capacity of NPs widely used in consumer products and cosmetics (e.g., zinc oxide, ZnO, and titanium dioxide, TiO_2_, present in sunscreens) or in therapeutic dermatological formulations (e.g., solid lipid NPs, SLN).

Uptake studies in these systems consistently demonstrate that while an intact stratum corneum represents an insurmountable barrier for most NPs with dimensions exceeding 20-30 nm, mechanical alterations of the tissue or specialized formulations (e.g., ultra-flexible NPs or transfersomes) manage to penetrate through skin appendages (hair follicles and sweat glands) or localize within the viable layers of the epidermis. This allows researchers to quantify both the efficacy of drug delivery and the risk of potential systemic toxicity or contact dermatitis induced by nanomaterials.

#### 7.1.4. Organ-on-a-Chip Platforms: Recapitulating Tumor Vasculature and Microenvironment Dynamics

Organ-on-a-chip (OoC) platforms integrate 3D cell biology with microfluidics, overcoming the lack of dynamic perfusion and shear stress in static spheroids. PDMS microchannels separate a 3D tumor compartment from an HUVEC-lined vascular channel [287], enabling controlled study of NPs adhesion under flow, trans-endothelial extravasation, and immune-cell co-perfusion for evaluating both immunotherapeutic efficacy and MPS-mediated clearance.

For the evaluation of NPs, tumor-on-a-chip models allow researchers to recreate the dynamic conditions of the tumor microenvironment: (A) Replication of Fluidic Flows: They facilitate the dynamic introduction of NPs within the microfluidic flow, simulating intravenous administration. This enables real-time study of nanocarrier adhesion to endothelial walls under the influence of blood shear stress. (B) Modeling Extravasation: They permit the quantitative analysis of trans-endothelial extravasation—specifically, the capacity of the nanocarrier to cross the fenestrated endothelial junctions typical of tumor vasculature and actively penetrate the adjacent 3D tumor mass. (C) Immune System Integration: They allow the co-perfusion of immune cells, providing a robust tool to evaluate both the efficacy of immunotherapeutic nanoformulations and the rate of non-specific clearance driven by mononuclear phagocytes under continuous flow conditions.

#### 7.1.5. Comparison of 2D vs. 3D Model Predictive Value: Advantages, Limitations, and Translational Relevance

The rational adoption of an experimental model requires a profound understanding of the balance between its biological complexity and its actual translational predictivity. Table 3 summarizes the main comparative parameters between traditional 2D systems and advanced 3D models for nanoparticle-based cancer drug delivery studies.

### 7.2. In Vivo Preclinical Studies

#### 7.2.1. Murine Tumor Models: Subcutaneous, Orthotopic, Patient-Derived Xenografts (PDX)

Despite the rapid progress of 3D in vitro models, final preclinical validation of a nanoformulation requires transitioning to in vivo animal models to evaluate complex pharmacokinetics and systemic toxicity. Murine tumor models represent the standard of choice and are divided into three main categories:

1. Subcutaneous (Ectopic) Models: These involve the inoculation of tumor cells (either human cells into immunodeficient nude mice, or murine cells into syngeneic immunocompetent mice) into the subcutaneous space of the animal’s flank. They are the most widely used models due to their technical simplicity and the ease of monitoring tumor volume via direct caliper measurements. However, they possess low clinical relevance because the subcutaneous microenvironment differs radically from the original tissue site, altering vascularization and drug response [292].
2. Orthotopic Models: Tumor cells are implanted directly into their organ of origin (e.g., breast cancer cells into the mammary fat pad, intracranially for glioblastoma). These models faithfully recreate the native vasculature, nutrient availability, and interactions with the organ stroma, enabling accurate studies of metastatic processes. They require advanced in vivo imaging modalities (e.g., IVIS bioluminescence, micro-CT, or MRI) to monitor internal tumor progression [287].
3. Patient-Derived Xenografts (PDX): These represent the frontier of translational in vivo oncology. Intact tumor tissue fragments, harvested directly from a patient’s surgical resection, are implanted into immunodeficient mice without a prior in vitro cell dissociation phase. PDX models preserve the original histological architecture, cellular heterogeneity, tumor stroma, and genetic/mutational profile of the human patient. They constitute the most predictive tool for validating the efficacy of nanoformulations targeted against specific cellular subpopulations (e.g., cancer stem cells), although they are constrained by high maintenance costs, long tumor engraftment timelines, and the necessity of operating in models lacking a fully functional immune system.

#### 7.2.2. Biodistribution, EPR Effect, and Tumor Accumulation

After intravenous injection, passive tumor targeting has classically relied on the EPR effect: leaky tumor vasculature allows 20–150 nm nanocarriers to extravasate and accumulate, while impaired lymphatic drainage limits clearance [287,292,298]. However, EPR is substantially countered by MPS-mediated hepatosplenic clearance following opsonization and protein-corona formation. PEGylation remains the principal strategy to reduce opsonin binding, extend circulation half-life, and maximize EPR-based accumulation. Critically, high whole-tumor accumulation, as measured by bulk biodistribution assays (e.g., %ID/g), should not be equated with homogeneous intratumoral distribution or with effective delivery to poorly vascularized, hypoxic tumor regions. As discussed in Section 7.1, nanocarriers larger than approximately 100 nm tend to remain confined to the peripheral, well-perfused proliferative rim of multicellular tumor spheroids because of dense extracellular matrix and elevated interstitial fluid pressure, leaving hypoxic core regions comparatively under-dosed even when total tumor uptake is high. This distinction between whole-tumor accumulation and true intratumoral distribution is essential for correctly interpreting biodistribution data.

#### 7.2.3. Toxicological Profiling: Hematological and Histopathological Evaluations

In vivo toxicological profiling combines hematology (complete blood count for RBC/WBC/platelet abnormalities) with clinical biochemistry to detect immunotoxic, anemic, or organ-specific adverse effects following acute or chronic nanoformulation administration [287].

Definitive safety assessment relies on ex vivo histopathology of liver, spleen, kidney, lung, heart, and brain, screening for inflammatory infiltration or granuloma formation, hepatocyte necrosis, renal tubular damage, and pulmonary fibrosis or micro-embolism associated with nanocarrier accumulation.

## 8. Multimodal Nanoplatforms for Theranostics and Physiological Monitoring

**Figure 4:**
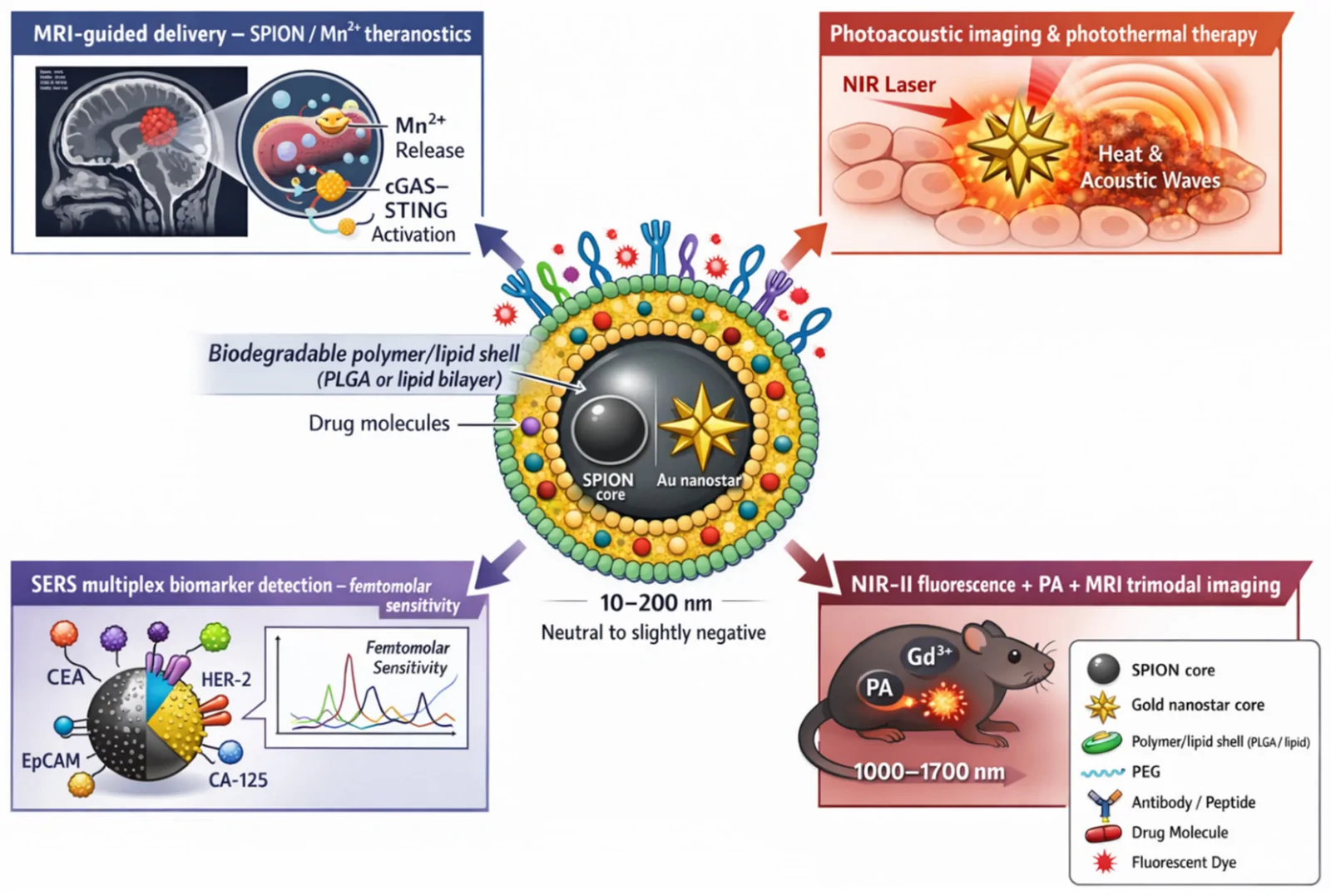
Modular design of an ideally multifunctional theranostic nanoplatform (10–200 nm) integrating SPION/Mn^2+^-based MRI guidance with cGAS–STING activation, gold nanostar-mediated photoacoustic imaging and photothermal therapy, SERS-based femtomolar multiplex biomarker detection, and NIR-II fluorescence for trimodal PA/MRI/NIR-II in vivo imaging. *During the preparation of this figure, the author(s) used FigureLabs to generate the graphical illustration of the modular multifunctional theranostic nanoplatform, based on the authors’ original concept, structural layout, and scientific content. The authors have reviewed and edited the output and take full responsibility for the content of this figure*.

Theranostics, combining therapy and diagnostics in one nanoconstruct, addresses a core limitation of conventional oncology workflows, where diagnosis, treatment, and response assessment are sequential and may span weeks of ineffective therapy. By collapsing this timeline, theranostic nanoplatforms enable real-time, adaptive treatment personalization, as shown by Saladino et al.’s 2025 MRI-fluorescence dual-mode platform tracking NPs accumulation and vascular disruption in orthotopic glioblastoma [301]. Recent advances have led to the development of complex nanosystems designed for precision medicine, as depicted in Figure 4. These systems exemplify multimodal theranostics, as they integrate SPION/Mn^2+^-based MRI guidance with cGAS–STING activation for cancer immunotherapy while simultaneously leveraging gold nanostar-mediated photoacoustic imaging and photothermal therapy, SERS-based femtomolar multiplex biomarker detection, and NIR-II fluorescence for comprehensive trimodal in vivo imaging.

While the modular integration of MRI/Mn^2+^ guidance, cGAS–STING immunoactivation, gold nanostar-mediated photoacoustic/photothermal function, SERS-based biomarker detection, and NIR-II fluorescence in Figure 4 illustrates the technical ceiling of current theranostic engineering, this same complexity makes explicit the trade-offs discussed throughout this review. Combining five or more functional components within a single 10–200 nm particle multiplies the degrees of freedom in synthesis, creating major batch-to-batch reproducibility and quality-control challenges; each additional surface-bound moiety (targeting ligand, imaging label, stimuli-responsive linker) can alter protein-corona composition, circulation half-life, and biodistribution in ways not readily predictable from single-component behavior, complicating pharmacokinetic and toxicological characterization. From a regulatory standpoint, each functional module (drug, device, diagnostic) may fall under a different evaluation pathway, and neither the FDA nor the EMA currently provides a harmonized framework for multi-component theranostic combination products (Section 9.7). Figure 4 should therefore be interpreted as an integrative design template illustrating the outer boundary of current engineering feasibility, rather than a construct that is presently synthesizable, reproducible, or evaluable as a single clinical entity; simpler, application-specific combinations of two to three functional modules are more likely to advance toward translational studies in the near term.

### 8.1. Design Principles of Theranostic Nanoplatforms

An effective theranostic nanoplatform must combine appropriate size/charge (10-200 nm, near-neutral) for EPR-based accumulation, an imaging component sensitive at clinically acceptable doses, controlled site-specific release, and a safety profile for repeated dosing, balanced against the “jack of all trades, master of none” risk that multifunctionality compromises individual component performance.

The most successful theranostic platforms use modular core-shell architecture: an inorganic core (SPION, gold, upconversion NPs) provides imaging contrast, a biodegradable shell (PLGA, PLA, lipid bilayer) carries the drug, and surface functionalization adds stealth/targeting. Combining modalities compensates for individual limitations, MRI (deep penetration, low sensitivity), fluorescence (high sensitivity, limited depth), photoacoustic (intermediate-depth resolution), and SERS (molecular specificity) [302].

### 8.2. MRI-Guided Drug Delivery with SPION-Based Theranostics

SPIONs are the most clinically advanced inorganic nanoplatform, but their regulatory status is heterogeneous and should not be read as validation of SPION-based theranostic drug delivery. Ferumoxytol (Feraheme) is an FDA-approved iron-replacement therapy for chronic kidney disease, used off-label as an MRI contrast agent; ferucarbotran (Resovist) is an imaging contrast agent approved in the EU and Japan (though discontinued or of limited availability in most markets) for liver MRI. Neither is an approved drug-delivery or theranostic product, and no SPION-based cancer theranostic system currently has regulatory approval. Within this context, repurposed ferumoxytol shows MRI accumulation that correlates quantitatively with microscopic nanoparticle distribution in orthotopic glioblastoma models [301], illustrating imaging-tracking feasibility rather than clinical efficacy as a drug carrier; ultrasmall SPIONs (<5 nm) generate T1 contrast with renal clearance, addressing long-term biodistribution concerns at the preclinical stage [303,304].

Mn^2+^-based NPs provide complementary T_1_ contrast while activating the cGAS-STING pathway upon release in the tumor microenvironment, representing a further preclinical—not clinically validated—theranostic strategy [305,306,307,308].

### 8.3. Photoacoustic Imaging and Photothermal Therapy Nanoplatforms

Photoacoustic imaging converts pulsed-laser thermoelastic expansion into acoustic images; gold nanorods/nanostars and carbon nanotubes are leading PA contrast agents. IO@MnO_2_@DOX hybrids exemplify TME-responsive PA/MRI theranostics: the IO core gives T2 contrast/hyperthermia, while GSH-triggered MnO_2_ degradation releases both Mn^2+^ (T1 contrast) and DOX for combined chemo/magnetothermal therapy and dual imaging [309].

Gold nanostars are exceptionally versatile theranostic platforms [310,311]: their multi-tip geometry generates intense NIR plasmon fields ideal for SERS, PA imaging, CT contrast, and PTT in a single surfactant-free particle [312]. Antibody/photosensitizer-loaded AuNS achieved simultaneous tumor delineation and PDT/PTT with complete ablation at laser fluences below skin safety limits [313,314].

### 8.4. SERS-Active NPs for Cancer Biomarker Detection and Drug Monitoring

SERS exploits electromagnetic field enhancement in nanometer gaps between metallic nanostructures, amplifying Raman signal 10^8^–10^14^-fold, approaching single-molecule sensitivity. Established expertise in Janus silver nanoparticle SERS probes provides a competitive platform for next-generation theranostic SERS agents [315].

SERS NPs with Raman-reporter labels (4-aminothiophenol, DTNB, rhodamine 6G, malachite green) conjugated to antibodies/aptamers enable multiplexed detection of cancer biomarker panels (CEA, Her-2, EpCAM, CA-125) at femtomolar sensitivity in a single measurement [316], offering minimally invasive liquid-biopsy detection complementing ELISA/PCR [317].

### 8.5. NIR-II Fluorescence and Multimodal Imaging Nanoplatforms

NIR-II (1000–1700 nm) fluorescent probes offer deeper tissue penetration and lower background than NIR-I agents [318], with SWCNTs, rare-earth-doped UCNPs, Ag_2_S/Ag_2_Se quantum dots and organic NIR-II fluorophores explored as imaging agents. He et al.’s folate-targeted apoferritin platform co-loading IR1061 dye and paclitaxel achieved pH- and NIR-II-laser-triggered synergistic photothermal-chemotherapy in a 4T1 model [319,320].

Trifunctional MRI/PA/NIR-II platforms are increasingly tractable: Hu et al. engineered Gd-chelated semiconducting polymer NPs (PFTQ-PEG-Gd) whose D-A back-bone provides PA imaging and photothermal activity, NIR-II fluorescence, and Gd^3+^-mediated T1 MRI contrast in a single construct, demonstrating simultaneous PA, NIR-II, and MRI enhancement with NIR-triggered tumor photothermal suppression in 4T1-bearing mice [321].

### 8.6. Wearable and Implantable Nanosensor-Based Physiological Monitoring

Nanoparticle-functionalized wearable/implantable biosensors enable continuous, minimally invasive monitoring of cancer biomarkers. Gold/silver NPs -based microneedle electrodes, functionalized with antibodies or aptamers, have achieved label-free interstitial-fluid detection of CEA and ErbB2 at clinically relevant concentrations and proof-of-concept multiplexed detection of CEA, AFP, CA-125, and CA-153, though translation to continuous wearable formats remains under investigation [322,323,324,325].

SERS-based implantable sensors offer complementary label-free specificity: subcutaneous AgFON substrates enabled the first transcutaneous SERS glucose measurements, later extended to 17-day continuous monitoring, and to flexible wearable SERS patches for sweat-based lactate, urea, glucose, uric acid, and tyrosine profiling, an approach directly relevant to cancer-metabolite (e.g., lactate/Warburg effect) monitoring [326,327,328,329].

## 9. Artificial Intelligence and Machine Learning in Nanoparticle Design

Nanoparticle optimization involves a formidable multidimensional parameter space (polymer weight, drug-to-polymer ratio, solvent, preparation method, surface chemistry) that is difficult to model intuitively; classical Design-of-Experiment approaches remain limited for non-linear interactions, motivating AI/Machine-Learning (ML) approaches that learn structure-property-activity relationships directly from data [330].

**Figure 5:**
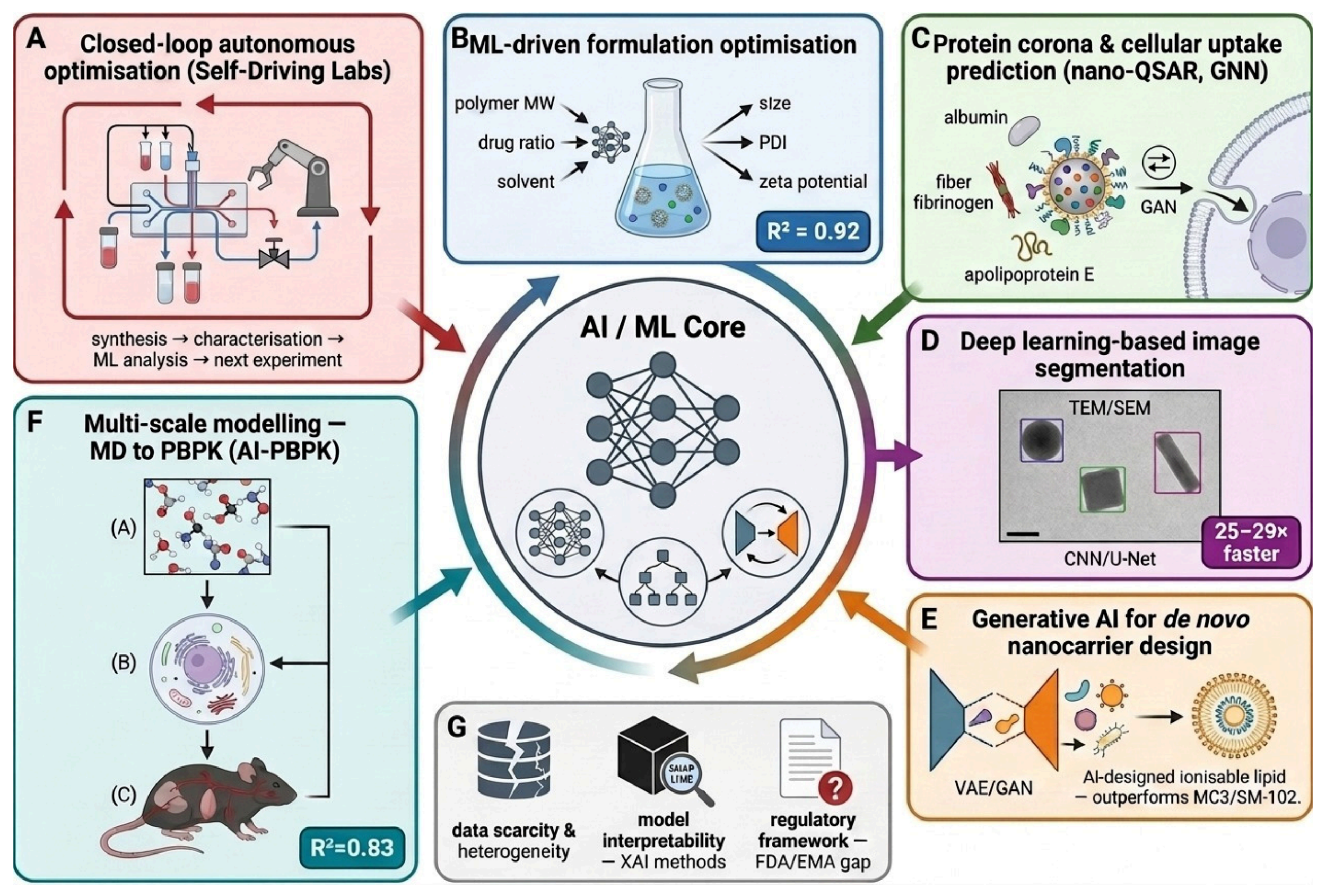
Overview of AI/ML applications across the nanomedicine pipeline, organised around a shared AI/ML core. (**A**) Closed-loop autonomous optimisation (self-driving labs). (**B**) ML-driven formulation optimisation of size, PDI, and zeta potential (R^2^ = 0.92). (**C**) Protein corona and cellular uptake prediction via nano-QSAR/GNN models. (**D**) Deep learning-based image segmentation of TEM/SEM micrographs (CNN/U-Net; 25–29× faster). (**E**) Generative AI (VAE/GAN) for de novo nanocarrier design. (**F**) Multi-scale modelling from molecular dynamics to PBPK simulation (R^2^ = 0.83). (**G**) Key challenges: data scarcity/heterogeneity, model interpretability (XAI), and regulatory gaps (FDA/EMA). *During the preparation of this figure, the author(s) used FigureLabs to generate the graphical illustration of the AI/ML-driven nanoparticle development pipeline, based on the authors’ original concept, structural layout, and scientific content. The authors have reviewed and edited the output and take full responsibility for the content of this figure*.

The pharmaceutical and nanomedicine literature has witnessed an exponential growth in AI/ML publications since 2020, with applications spanning every stage of the NPs development pipeline: from in silico material design and formulation optimization to protein corona prediction, cellular uptake modeling, automated characterization, pharmacokinetic prediction, and clinical dosing optimization (Figure 5). Wang et al. in a comprehensive review categorized ML applications across all stages of nanoparticle drug delivery, from synthesis parameter prediction to in vivo biodistribution modeling, and provided critical assessment of each approach’s maturity level and translational readiness [330].

Concretely, AI/ML contributes to nanoparticle design by (i) predicting the relationship between synthesis parameters and resulting physicochemical properties to reduce trial-and-error formulation screening (Section 9.1); (ii) modeling nano–bio interface behavior, including protein-corona composition and cellular uptake, to guide surface engineering (Section 9.2); (iii) automating microscopy-based characterization for higher-throughput quality control (Section 9.3); (iv) generating novel carrier materials and ligand structures beyond existing libraries (Section 9.4); (v) linking molecular-scale simulations to whole-body pharmacokinetic prediction (Section 9.5); and (vi) closing the design–synthesis–characterization loop within autonomous, self-driving experimental platforms (Section 9.6). Throughout this section, findings are explicitly distinguished by evidentiary maturity: in-distribution, retrospective computational performance metrics; single-study preclinical (in vitro or animal) demonstrations; and evidence consolidated across multiple independent studies or meta-analyses are not equivalent and are flagged as such, following the same standard applied to the NanoMAP/Tao et al. comparison in Section 9.6.

### 9.1. Machine Learning for Formulation Optimization and Property Prediction

ML’s most immediately practical nanomedicine application is automated formulation optimization and in silico property prediction: artificial neural networks and deep learning now accurately map the complex, non-linear relationship between synthesis parameters and resulting hydrodynamic diameter, PDI, and zeta potential, previously reliant on costly trial-and-error or classical statistical design. Meta-analyses show models trained on polymer attributes (molecular weight, hydrophobicity, viscosity) reliably predict carrier size distribution, with deep neural networks trained on nanomedicine repositories achieving R2 up to 0.92 for delivery efficiency and biological behavior; these performance metrics reflect in-distribution accuracy on the specific datasets and repositories evaluated and should not yet be read as evidence of externally validated, generalizable predictive power across independent nanomedicine libraries [331].

### 9.2. Machine Learning for Nano–Bio Interface Prediction

The nano–bio interface, encompassing protein corona formation, cell membrane interactions, endosomal trafficking, and cytotoxicity, is the critical determinant of in vivo NPs performance, yet it is notoriously difficult to predict from physico-chemical descriptors alone. ML approaches are making rapid progress on this challenge, exploiting proteomics data from carries–serum incubation experiments and high-content imaging data from cellular uptake assays to build predictive models of increasing accuracy [330,331].

ML models trained on proteomic (LC-MS/MS) data now predict protein-corona composition from carriers surface chemistry and size, identifying which plasma proteins preferentially adsorb and guiding rational surface engineering (PEGylation, zwitterionic coatings) to reduce opsonization [331].

At the cellular level, quantitative structure–activity relationship (QSAR) models at the nano–bio interface (nano-QSAR) correlate NPs descriptors with uptake efficiency in specific cell lines. Graph neural networks (GNNs), which encode NPs surface chemistry as molecular graphs, have outperformed classical descriptor-based QSAR models in predicting cellular uptake across a diverse library of polymeric NPs in cancer cell lines, in the single retrospective study reported to date, suggesting that the graph representation captures important structural motifs that linear descriptors miss.

### 9.3. Deep Learning for Automated Nanoparticle Characterization

Transmission electron microscopy (TEM) and scanning electron microscopy (SEM) provide essential information on NPs size, morphology, and structure, but manual image analysis remains time-consuming, subjective, and poorly scalable to the high-throughput workflows increasingly demanded in nanoparticle development pipelines. The adoption of deep learning architectures for automated microscopy image analysis represents one of the most significant methodological advances in nanomaterial characterization in recent years.

Convolutional neural networks (CNNs) variants (U-Net, U-Net++, Mask R-CNN) are now standard for automated TEM/SEM segmentation, extracting particle size and shape distributions from 2D/3D micrographs. U-Net++/ResNet34 workflows distinguish spherical, cubic, and rod-shaped particles, even overlapping ones, validated across SiO_2_, Au, Ag, and ZnO nanoparticle datasets [332].

Fully automated pipelines integrating semantic segmentation models with size measurement algorithms have been validated on datasets of 1701 two-dimensional particle images, demonstrating high accuracy even in the presence of image noise or blurriness, with results comparable to expert manual annotations [333]. A further advance is represented by multi-output convolutional approaches: multiple-output CNNs (MO-CNN) enable the simultaneous detection, localization, and segmentation of NPs from TEM images, providing both the spatial coordinates of each particle and the boundary profiles required for size estimation via a modified Hough transform [334].

From a high-throughput perspective, automated segmentation and acquisition coordinate generation pipelines have demonstrated a 25- to 29-fold reduction in total analysis time compared with conventional protocols, achieving a 96% success rate on datasets of over 900 expert-validated high-resolution images. These results confirm that automated TEM analysis is now a viable option for quality control in NPs manufacturing at industrial scale [335].

A complementary and promising direction involves integrating AI-driven formulation design with automated, high-throughput experimental platforms to accelerate nanocarrier development.

Similarly, deep neural network (DNN)-based approaches have been introduced to enhance Dynamic Light Scattering (DLS) performance in characterizing multimodal particle size distributions, overcoming the limitations of conventional autocorrelation function fitting methods, which often fail to capture the complexity of real heterogeneous samples. The application of neural networks to DLS signal analysis has yielded substantial speed improvements over traditional Lorentzian fitting, while maintaining high accuracy across a broad range of particle sizes [336].

Collectively, these advances demonstrate that deep learning and machine learning are fundamentally reshaping NPs characterization workflows. By automating image segmentation, enabling high-throughput quality control, and extending predictive capabilities to accessible optical techniques such as DLS and UV-vis spectroscopy, these approaches substantially reduce both the time and expertise burden associated with conventional analysis. As training datasets expand and model architecture continue to evolve, fully automated, real-time characterization pipelines are expected to become standard practice in both research and industrial carriers’ development.

### 9.4. Generative AI for De Novo Nanocarrier Material Design

Generative AI models—variational autoencoders (VAEs), generative adversarial networks (GANs), and transformer-based large language models (LLMs) fine-tuned on chemical databases—are moving beyond the optimization of known formulations to the generation of entirely new nanocarrier materials with user-specified properties. This represents a paradigm shift from data-interpolation to data-extrapolation: rather than finding the best formulation within the experimental space already explored, generative models propose structures outside that space, potentially discovering materials with properties unattainable by existing compounds.

The most compelling application of generative AI in drug delivery is designing ionizable lipids for LNP-mRNA delivery: ML models trained on structure-transfection datasets proposed candidates that outperformed benchmark lipids (MC3, SM-102) in vivo in murine models, with AI-optimized lipids achieving organ-targeted (including hepatic) mRNA expression exceeding clinical controls at the preclinical stage [337,338]. Bhujel et al.’s 2025 review of AI-driven RNA-LNP vaccine design confirmed such integration reduces development timelines from years to months [339].

For polymeric nanocarriers, VAE-based generative models have demonstrated the ability to design novel polymer architectures with targeted structural properties, showing that the latent space learned by generative models encodes meaningful structure–property relationships exploitable for rational materials discovery [340]. Complementary machine learning frameworks applied to polymeric biomaterials—including PLGA-based systems—have further enabled prediction of degradation profiles and biocompatibility parameters relevant to drug delivery applications [341].

### 9.5. Multi-Scale Computational Modeling and AI-PBPK Integration

A complementary pillar of AI in nanomedicine is multiscale computational modeling—the integration of molecular dynamics (MD) simulations, coarse-grained models, and physiologically-based pharmacokinetic (PBPK) models into a cohesive computational framework that predicts nanoparticle behavior from atomic interactions to whole-body pharmacokinetics. Each scale provides insights inaccessible to the others, and their integration is essential for a mechanistic understanding of nanoparticle delivery that can guide clinical translation.

At the molecular scale, machine-learned force fields (SchNet, DimeNet++, NequIP, MACE) combine near-quantum accuracy with classical-force-field efficiency, enabling large-scale MD simulations of biomolecular and metallic nano systems [342,343]. Their direct application to drug-loaded lipid-bilayer/nano systems remains emerging but offers a promising route to predicting binding affinities and membrane-permeation barriers ahead of synthesis.

At the organism scale, AI-augmented PBPK models predict tumor accumulation from physicochemical descriptors. A representative QSAR–PBPK framework trained on a Nano-Tumor Database predicted delivery efficiency with R2 = 0.82–0.83 across 288 retrospectively compiled preclinical data sets, a performance reported in retrospective analyses and since confirmed and extended by subsequent AI–PBPK reviews incorporating SHAP-based feature attribution [344,345].

### 9.6. Self-Driving Laboratories and Autonomous Nanoparticle Optimization

The self-driving laboratory (SDL) represents the most ambitious convergence of robotics and AI in NPs science: closed-loop platforms that combine automated synthesis, inline characterization, and ML-guided experiment planning with minimal human intervention. Hickman et al. proposed NanoMAP, a nanomedicine-specific materials acceleration platform built on high-throughput nanoprecipitation, active learning for efficient dataset construction, and few-shot/meta-learning models to generalize across active agents; however, this remains a conceptual roadmap structured around three unmet milestones, not a validated operational system, and the article reports no quantitative performance outcomes (such as convergence times or fold-improvements in drug loading) [346].

A concrete, already-realized example of an SDL applied to NPs synthesis, cited within this same framework, is the platform of Tao et al., which integrated microfluidic synthesis with machine learning to autonomously optimize the synthesis conditions of metal (gold/silver) NPs based on their spectroscopic properties [347]. While this system targeted inorganic rather than polymeric drug-delivery NPs, it demonstrates that the building blocks proposed for nanomedicine-focused SDLs, microfluidic automation coupled with closed-loop ML optimization, are technically feasible and have already been deployed for related nanomaterial classes.

### 9.7. Current Limitations and Open Challenges

Despite the exceptional promise of AI in nanomedicine, a candid assessment of current limitations is essential for setting realistic expectations. The most fundamental challenge is data scarcity and heterogeneity: NPs datasets in the public domain are typically small and generated with heterogeneous protocols and reporting standards, severely limiting ML model generalizability beyond the training distribution. Harmonization initiatives such as the MIRIBEL reporting standard and adherence to FAIR (Findable, Accessible, Interoperable, Reusable) data principles are expected to play a central role in establishing reusable, trustworthy datasets across institutions and platforms, though broad community uptake remains a work in progress [348].

A second challenge is model interpretability: deep-learning models often function as “black boxes,” raising concerns for high-stakes applications like toxicity prediction. Explainable-AI methods (SHAP, LIME, attention mechanisms) are increasingly applied to render nanoparticle-property models interpretable [348,349].

Regulatory acceptance of AI-assisted design remains unresolved: neither the FDA nor the EMA has issued guidance specific to AI-designed nanomedicines. Existing frameworks address either AI/ML-based medical device software (FDA, 2021—“Artificial Intelligence/Machine Learning (AI/ML)-Based Software as a Medical Device (SaMD) Action Plan” (https://www.fda.gov/medical-devices/software-medical-device-samd/artificial-intelligence-and-machine-learning-aiml-enabled-medical-devices, accessed on 15 August 2016)) or nanomaterial-containing drug products generally (FDA, 2022—“Drug Products, Including Biological Products, that Contain Nanomaterials—Guidance for Industry” (https://www.fda.gov/regulatory-information/search-fda-guidance-documents/drug-products-including-biological-products-contain-nanomaterials-guidance-industry, accessed on 15 August 2016)), leaving AI-driven formulation design without dedicated regulatory guidance.

## 10. Challenges, Clinical Translation, and Future Perspectives

Despite remarkable progress in the design of NPs and biomolecule-based drug delivery systems, their successful clinical translation remains constrained by several scientific, technological, regulatory, and manufacturing challenges. During the last five years (2022–2026), the focus of research has progressively shifted from demonstrating proof-of-concept efficacy toward improving reproducibility, scalability, regulatory compliance, and personalized nanomedicine.

### 10.1. Scalability of NPs Manufacturing: From Laboratory Proof-of-Concept to GMP-Compliant Production

Manufacturing scalability remains a primary bottleneck, since laboratory-scale formulations often fail to preserve critical quality attributes (particle size, polydispersity, surface chemistry, drug loading) upon industrial translation. Continuous manufacturing, microfluidic synthesis, and process analytical technologies (PAT) improve reproducibility under GMP conditions and are increasingly emphasized by regulators as prerequisites for approval.

Clinical translation ultimately depends on robust, scalable manufacturing: although thousands of nanoformulations show preclinical promise, few reach approval, largely because bench-scale synthesis rarely meets industrial requirements. This has shifted attention from efficacy alone toward quality-by-design (QbD) and standardized, regulatory-oriented process development [350].

Batch-to-batch reproducibility is particularly challenging, as NPs’ critical quality attributes are highly sensitive to small variations in solvent composition, mixing rate, temperature, and purification. Many promising formulations fail during technology transfer owing to insufficient process robustness or operator-dependent manual steps [351].

A translation-oriented approach to engineered LBNPs for advanced tumor targeting has demonstrated that the future clinical success of oncological nanomedicines depends less on demonstrating preclinical efficacy and much more on overcoming bottlenecks related to industrial scalability, designing carriers specific to each therapy, and establishing consistent global regulatory pathways.

Joyce et al. identify manufacturing as a central pillar of the DELIVER (Design, Experimental, Long-term safety, Industrialization, Validation, Evaluation, and Regulatory) translational framework, arguing that scalability should be addressed from the earliest design stage rather than after proof-of-concept, with manufacturing, regulatory strategy, and preclinical validation evolving in parallel rather than sequentially [350].

An equally important aspect concerns compliance with GMP requirements. Unlike conventional small-molecule pharmaceuticals, nanomedicines require extensive physicochemical characterization because multiple structural parameters collectively determine their biological behavior. Consequently, manufacturing processes must be capable of consistently controlling not only drug content but also NPs’ architecture, surface chemistry, impurity profiles, sterility, endotoxin levels, and long-term stability. The industry perspective presented by Clogston et al. highlights that Chemistry, Manufacturing, and Controls (CMC) has become one of the most demanding components of regulatory submissions for nanomedicines. Modern regulatory expectations increasingly require comprehensive characterization of CQAs using orthogonal analytical methods, validated release assays, stability-indicating methodologies, and rigorous process validation throughout the product lifecycle. Furthermore, manufacturers are encouraged to establish robust control strategies capable of identifying critical process parameters that directly affect NPs quality and performance [351].

Khairnar et al. (2022) discussed some conventional methods and the rationale for large-scale production of solid lipid NPs (SLNs), including high-pressure homogenization (HPH), hot melt extrusion coupled with HPH, microchannels, nanoprecipitation using static mixers, and microemulsion-based methods [352]. These scale-up technologies enable the possibility of commercialization of SLNs. Furthermore, ongoing studies indicate that these technologies will eventually reach the pharmaceutical market [352]. In the same year, Operti et al. showed that an inline sonication process could be transferred from batch lab preparation to industrial-scale production of PLGA nanovaccines, while preserving colloidal, functional, and toxicological profiles [353].

Recent manufacturing studies (2024–2025) directly relevant to oncology include a micro-spray-reactor/double-emulsion process for scalable PLGA NPs production [354] and sequential flash nanocomplexation/nanoprecipitation for low-batch-variation DOX-loaded PEG-b-PLGA NPs purified by tangential flow filtration [355].

Therefore, the current thread is the intensification of mixing or continuous processing: inline sonication, microfluidic rapid mixing, channel enlargement, flash nanoprecipitation, and damped continuous-flow nanoprecipitation. In the context of research and development, a number of laboratory-scale flow processes have been utilized with the objective of enhancing the scalability of nanocarriers. These processes include spray drying and ultrasonication, which have been employed to facilitate the technology transfer [356,357].

Flow-based platforms outperform bulk mixing by offering precise control over solvent exchange, nucleation, and self-assembly, yielding more uniform, reproducible NPs with reduced operator-dependent variability and a clearer scale-up path via parallelization—particularly valuable for lipid, polymeric, and nucleic-acid nanocarriers where formulation parameters strongly affect biological activity [358].

Cao et al. improved continuous nanoprecipitation scalability by integrating pulsation-dampening into microfluidic devices, ensuring stable flow and uniform particles at scale [359], while Bonacorsi et al. increased liposome throughput to 30 L h-1 via a channel-enlargement strategy without compromising size or polydispersity [360]. Together these studies reinforce continuous, automated, QbD-driven manufacturing as the paradigm for industrial translation. Economic and regulatory pressures are further pushing the field toward simpler, more cost-effective formulations, since clinical success depends as much on manufacturability and supply-chain robustness as on biological sophistication [361].

Future advancements in the field of cancer nanomedicine must entail the incorporation of scalable manufacturing methodologies as an integral component of the initial design of NPs, rather than considering production as a subsequent consideration. The utilization of contemporary technologies provides a pragmatic approach to surmounting the entrenched obstacles impeding clinical translation. It is evident that the subsequent generation of effective nanomedicines will be characterized not only by their reproducible, economical, and compliant large-scale production, but also by their medical efficacy [350].

### 10.2. From Bench to Clinic: Biological Barriers and Long-Term Safety Considerations

Clinical translation depends not only on payload encapsulation/release but on overcoming biological barriers while maintaining long-term safety. Recent studies (2022–2026) attribute the gap between preclinical promise and clinical success largely to incomplete understanding of nanomaterial–biology interactions; research has consequently shifted from optimizing physicochemical properties alone toward designing carriers adaptable to individual patient physiology and tumor heterogeneity [350,358,362].

NPs rapidly acquire a protein corona after intravenous administration that redefines biological identity and varies with patient disease state and metabolism, partly explaining variable clinical performance [358]. Opsonization drives MPS clearance; PEGylation mitigates this but can trigger accelerated blood clearance or anti-PEG antibodies, prompting alternative stealth strategies (zwitterionic polymers, biomimetic membranes, albumin coatings) [350,358]. Because EPR varies substantially across tumors and patients, the field is shifting toward active multi-targeting ligand strategies combined with stimuli-responsive, combination-therapy release [361]; biomimetic membrane-coated carriers offer a complementary route inheriting endogenous immune evasion [350].

While improving delivery efficiency remains a primary objective, long-term safety has become an equally important consideration in nanomedicine development. Unlike conventional low-molecular-weight drugs, NPs possess unique physicochemical characteristics that may elicit nano-specific biological responses not readily predicted by standard toxicological assays. Immune-related adverse effects have emerged as one of the principal safety concerns, including complement activation-related pseudoallergy (CARPA), cytokine release, inflammasome activation, oxidative stress, and unintended modulation of innate or adaptive immune responses. These effects are influenced by particle composition, size, morphology, surface chemistry, and degradation products, underscoring the necessity for comprehensive immunotoxicology evaluation during preclinical development [351].

Long-term biodistribution and clearance remain unresolved: while lipid and biodegradable polymeric carriers are metabolized, inorganic NPs (gold, silica, iron oxide, quantum dots) may persist in the reticuloendothelial system, raising concerns over chronic inflammation, fibrosis, and delayed toxicity. Recent reviews therefore advocate incorporating long-term pharmacokinetic and repeated-dose toxicity studies from early development rather than relying on acute endpoints alone [350,358]. Genotoxicity is a related concern: although most approved nanomedicines show favorable safety profiles, some inorganic NPs have been associated with DNA damage, mitochondrial dysfunction, and oxidative stress under specific exposure conditions, with effects often depending more on physicochemical characteristics than composition, motivating integrated testing strategies combining in vitro, organ-on-chip, computational, and multi-omics approaches.

Overcoming biological barriers increasingly relies on combining active targeting, combination therapy, and stimuli-responsive release: folate-targeted dual-drug nanoliposomes improved tumor uptake with pH-triggered release [363]; DoE-optimized PLGA NPs penetrated glioblastoma and defeated multi-drug-resistant breast cancer [364,365]; and such formulations, including new LNPs, reduce cardio-/nephrotoxicity while boosting efficacy [366]. This has given rise to Safe-by-Design, integrating safety considerations (materials, architecture, biodegradability, manufacturing, immunocompatibility, regulation) from the earliest design stage, aligned with Quality-by-Design principles [367].

Next-generation nanocarriers are moving toward multifunctional platforms addressing several biological barriers simultaneously, improving tumor-specific accumulation while reducing systemic toxicity. Future systems will likely combine biomimetic surface engineering, programmable responsiveness, multi-targeting, and patient-specific profiling, supported by advanced biological models, AI, and precision medicine to improve predictability of both efficacy and safety.

Regulatory frameworks continue to evolve within existing pharmaceutical structures rather than dedicated legislation: the FDA’s 2022 nanomaterial guidance emphasizes risk-based, critical-material-attribute characterization, while the EMA has expanded reflection papers covering liposomes, polymeric micelles, and surface-coated NPs. The MIRIBEL framework promotes standardized reporting of nanoparticle characterization and biological assays, expected to improve interlaboratory comparability and regulatory evaluation (U.S. Food and Drug Administration).

Experience with nanomedicines (Doxil/CAELYX, Onpattro, Comirnaty, Abraxane, AmBisome) shows that controlled micromixing, solvent displacement, and QbD/PAT control are the most reusable manufacturing levers, with scale-up remaining the principal translational barrier [195]. These platforms confirm that clinical success depends less on structural complexity than on reproducible manufacturing, well-defined quality attributes, and clinically meaningful benefit [368,369].

### 10.3. The Paradigm Shift: Sustainability Considerations and Innovations in Personalized Nanomedicine

Sustainability is emerging as an important design criterion for nanocarriers in cancer drug delivery, complementing traditional objectives such as therapeutic efficacy, safety, and clinical translatability. Green chemistry principles are progressively being incorporated into NP synthesis through solvent-free and aqueous-based processes, plant- and microorganism-mediated production, energy-efficient manufacturing, and the replacement of persistent inorganic materials with biodegradable polymers, lipids, proteins, and other naturally derived biomaterials [370]. It should be stressed that in vivo or environmental biodegradability of the final carrier is not, by itself, a measure of overall environmental footprint: reduced long-term patient accumulation and reduced post-disposal persistence are distinct from the cumulative solvent, energy, water, and reagent burden of synthesis and purification; from the requirements of sterile, GMP-compliant manufacturing, and cold-chain storage and transport; and from batch yield and by-product generation—any of which can offset the benefits of a biodegradable end material [371]. Rigorously evaluating this trade-off requires formal life-cycle assessment or standardized green-chemistry metrics (e.g., process mass intensity, E-factor, solvent/water use per gram of product, energy demand, purification yield) applied across the full product life cycle, from raw materials and carrier synthesis to manufacturing, clinical use, and end-of-life disposal; such data remain scarce for nanomedicine formulations specifically.

Collectively, these considerations indicate that future nanomedicines should be designed not only to maximize therapeutic performance but also to minimize environmental burden across the full life cycle. We identify nanomedicine-specific LCA and green-metric studies as a priority for future work: only once such data exist can claims of alignment with circular-bioeconomy or sustainable-manufacturing principles be substantiated for specific nanocarrier platforms, rather than asserted from material biodegradability alone. From a 2026–2030 perspective, the future of NP-mediated cancer therapy will likely depend on the convergence of several complementary innovations rather than incremental improvements in individual carrier systems. Personalized nanomedicine is expected to become one of the most transformative directions for cancer drug delivery. The paradigm of cancer nanomedicine is progressively evolving from standardized drug delivery platforms toward patient-tailored therapeutic systems, driven by the convergence of multi-omics technologies, biomarker discovery, and artificial intelligence (AI).

Integration of genomic, transcriptomic, proteomic, metabolomic, and spatial omics data with patient-specific biomarkers will enable selection of optimal nanocarriers according to tumor phenotype, immune status, vascular characteristics, and predicted therapeutic response. Recent studies demonstrate that the integration of omics sciences provides an unprecedented understanding of tumor heterogeneity, enabling the identification of molecular signatures that guide the rational selection of nanocarrier composition, targeting ligands, therapeutic payloads, and administration strategies [372,373]. Rather than relying on universal formulations, next-generation nanomedicines are increasingly designed according to patient-specific biomarker profiles, allowing optimization of receptor targeting, metabolic stability, immune compatibility, and therapeutic response. In particular, omics-guided analysis of cell surface receptors and metabolizing enzymes has been proposed as a powerful approach for selecting both targeting moieties and carrier materials, thereby minimizing interpatient variability and improving treatment efficacy [374].

Future nanomedicines are expected to integrate multifunctional targeting, programmable release, biomimetic materials, and AI-assisted design within harmonized regulatory frameworks, supported by GMP-compatible continuous manufacturing and standards such as MIRIBEL [367]. AI and machine learning are increasingly applied to formulation optimization, biodistribution prediction, and adaptive manufacturing, with Process Analytical Technologies (PAT) enabling real-time monitoring and correction of critical manufacturing parameters [358]. Predictive modeling of physicochemical properties, protein-corona formation, and biological performance, coupled with digital twins and patient-derived organoids, is expected to accelerate personalized formulation validation ahead of clinical use [375,376,377].

Despite these remarkable advances, several challenges remain, including the scarcity of standardized multi-omics datasets, limited model interpretability, data interoperability, and the absence of harmonized regulatory frameworks for AI-assisted nanomedicine development. Nevertheless, the integration of omics-informed patient stratification with AI-driven formulation design represents one of the most promising frontiers of precision oncology, paving the way for intelligent nanotherapeutics capable of dynamically adapting to individual disease biology and maximizing therapeutic efficacy while minimizing adverse effects.

Looking beyond current developments, the convergence of multi-omics profiling, AI-assisted formulation design, digital twin technologies, and adaptive regulatory frameworks may ultimately enable real-time optimization of nanotherapeutics according to dynamic patient biomarker profiles. Such bio-digital ecosystems could transform nanomedicine from a static drug delivery platform into an intelligent therapeutic system capable of continuously adapting treatment to disease evolution [378].

## 11. Conclusions

Across the platforms reviewed here, cancer nanomedicine shows a consistent pattern: technologies built on simple, well-characterized architectures and established manufacturing routes have reached the clinic, whereas mechanistically sophisticated or biologically derived platforms remain largely preclinical. Lipid-based nanocarriers are the clearest cross-platform success story, with liposomal and lipid-nanoparticle formulations (Doxil/CAELYX, AmBisome, Onpattro, Comirnaty) and albumin-bound paclitaxel (Abraxane) in routine clinical use and superparamagnetic iron oxide nanoparticles (Feraheme, Resovist) approved as an iron-replacement therapy and an imaging contrast agent, respectively—though neither as a drug-delivery or theranostic product. Among targeting peptides, the iRGD-derived cyclic peptide certepetide (CEND-1/LSTA1) is the most clinically advanced construct, currently in phase 1b/2a–2b trials in pancreatic and other solid tumors, illustrating that conventionally engineered, rationally designed peptides—rather than AI-generated ones—remain the primary route to clinical-stage targeting agents to date.

By contrast, most platforms discussed in this review remain preclinical. Polymeric nanoparticles beyond a handful of approved formulations, phytochemical- and polysaccharide-based nanocarriers, nucleic-acid delivery systems beyond LNP–siRNA/mRNA, stimuli-responsive and tumor-microenvironment-triggered systems, STING-agonist and other immunomodulatory nanoplatforms, exosomes/extracellular vesicles, cell-membrane-coated nanoparticles, and multimodal theranostic constructs have consistently shown improved physicochemical behavior, cellular uptake, or antitumor activity in vitro and in animal models but lack the reproducible manufacturing, standardized characterization, and controlled human trial data required for clinical translation.

Two categories of barriers explain this gap. Biologically, heterogeneous EPR-mediated accumulation, protein-corona formation, mononuclear-phagocyte-system clearance, and—for biomimetic carriers—partial loss and reorientation of native membrane components during processing all limit translation of preclinical efficacy into predictable human pharmacokinetics. From a manufacturing standpoint, batch-to-batch reproducibility, GMP-compliant scale-up, sterility and endotoxin control, and, for exosomes specifically, unresolved regulatory classification remain the dominant bottlenecks; as the platforms that have reached the clinic illustrate, manufacturing robustness—not structural sophistication—is generally the limiting step.

Artificial intelligence is best understood, at this stage, as an accelerator embedded within this design-and-manufacturing pipeline rather than a distinct therapeutic modality. It already contributes to formulation optimization, nano–bio interface prediction, automated characterization, generative material design, and multi-scale PBPK modeling, but remains constrained by data scarcity and heterogeneity, limited model interpretability, and the absence of dedicated regulatory guidance for AI-assisted nanomedicine design. Its realistic near-term role is to narrow the experimental search space and improve process control—not to replace mechanistic validation or clinical evidence generation.

For the next five years, priorities should include the following: (i) standardized, FAIR-compliant reporting of nanoparticle characterization and biological data (e.g., MIRIBEL) to make preclinical findings comparable and AI models generalizable; (ii) prospective, adequately powered clinical studies for the most mechanistically promising preclinical platforms (biomimetic carriers, phytochemical nanomedicines, TME-responsive and immunomodulatory systems), rather than continued proliferation of new preclinical formulations; (iii) life-cycle assessment and green-chemistry metrics to substantiate—rather than assume—the environmental benefits of biodegradable materials; (iv) harmonized FDA/EMA regulatory pathways for exosome-based, cell-membrane-coated, and AI-designed nanomedicines; and (v) integration of multi-omics patient stratification with AI-assisted formulation design to move from platform-level comparisons toward genuinely personalized nanomedicine.

## Figures and Tables

**Table 2:** Biomolecule- and natural-compound-based cancer drug delivery strategies: carriers, mechanisms, stimuli-responsive/theranostic/AI-guided features, and highest level of supporting evidence.

Compound/ Category	Delivery System	Mechanism/Target	Key Findings	Stimuli-Responsive/Theranostic/AI Feature	Highest Level of Evidence
Curcumin	Liposomes, polymeric NPs, SLNs/NLCs, micelles, dendrimers, cyclodextrin complexes, BPNPs	Solubilization; protection from hepatic metabolism; active targeting (FA, transferrin, antibodies, peptides)	Free plasma level ~2.30 µg/mL after 10 g oral dose; reduced tumor growth/weight in 18 rodent studies (2022 meta-analysis); LDH-nanocurcumin effective in vivo against lung cancer	Multifunctional platform; theranostic potential	Animal efficacy (in vivo, preclinical)
Lycopene	Nanoemulsions, SEDDS, liposomes, polymeric NPs, peptide nanomicelles	Protection from oxidative instability; enhanced intracellular uptake	~2.2-fold increase in intracellular accumulation with peptide nanomicelles; Tween 20 vs. PVA-stabilized PLGA NPs show different colloidal stability/retention	Thermoresponsive NPs with enhanced anticancer activity	Increased cellular uptake (in vitro)
Thymol	Solid lipid NPs, metallic nanoplatforms	Overcomes volatility and low aqueous solubility	Higher cytotoxicity vs. free thymol on HT-29 cells; sustained release	Metallic nanoplatforms with imaging/photothermal function (theranostic)	In vitro cytotoxicity (cell-based)
Flavonoids/Anthocyanins	Nanoemulsions, liposomes, niosomes, polymeric NPs, BPNPs, nanogels	Protection from degradation; increased bioavailability	Encapsulated LYC: IC50 halved in a cell-free DPPH radical-scavenging assay; curcumin-casein complexes: half-life 8.8 → 340 min (~39-fold); fisetin liposomes: bioavailability up to 47-fold	Enhanced antioxidant effect via nanoencapsulation	Physicochemical/antioxidant assay only (cell-free; not anticancer efficacy)
Chitosan/ Alginate	Chitosan/alginate NPs, hydrogels	pH-sensitive protonation → selective release in acidic TME	Markedly reduced IC50 for gefitinib in lung cancer; high encapsulation efficiency for amygdalin	pH-responsive release in tumor microenvironment	In vitro cytotoxicity (cell-based, reduced IC50)
Carrageenan	κ-carrageenan/chitosan hydrogels; iota-carrageenan/β-cyclodextrin systems	Versatile carrier; limited intrinsic bioactivity	pH-responsive sunitinib release; enhanced methotrexate delivery	pH-responsive delivery system	Physicochemical improvement (in vitro release kinetics)
Fucoidan	P-selectin-targeted nanocarriers	VEGF/VEGFR suppression; STING–TBK1–IRF3 activation; PD-L1 downregulation	Improved tumor accumulation/efficacy of doxorubicin, paclitaxel, cisplatin; BBB crossing via caveolin-1-mediated transcytosis	Intrinsic immunomodulatory activity combined with carrier function	Animal efficacy (in vivo tumor models)
Hyaluronic acid (HA)	HA-functionalized NPs, nanocapsules	CD44 receptor targeting	Improved tumor-specific delivery of doxorubicin, icaritin, let-7b miRNA	Redox- (cystamine/GSH), pH- (DMA), photo- (azobenzene) responsive systems	Animal efficacy (in vivo tumor-specific delivery)
Other polysaccharides (galactose/glucose, alginate, pectin RG-I)	Nanomicelles, polymer conjugates	Receptor-specific targeting (hepatic ASGPR, macrophage mannose receptor, Galectin-3)	Hepato-specific and hepatocellular carcinoma delivery	Multiple receptor-targeting strategies	Preclinical efficacy (in vitro/animal, hepatic models)
Bacterial exopolysaccharides (dextran, pullulan, gellan gum, bacterial cellulose, levan, curdlan)	NPs, nanogels, hybrid hydrogels	Manufacturing scalability; intrinsic immunomodulation	Improved structural control via fermentation optimization	Integration into multifunctional theranostic platforms (delivery + imaging + immunomodulation)	Physicochemical improvement (manufacturing/structural characterization)
siRNA/miRNA/ASO/mRNA	Ionizable LNPs, polymeric NPs, dendrimers, exosomes, inorganic carriers	Endosomal protonation → membrane destabilization → cytoplasmic release	Clinical validation: patisiran (systemic LNP), COVID-19 mRNA vaccines; MRX34 trial failure (innate immune activation)	AI-assisted design of ionizable lipids/RNA sequences; SORT-LNP strategies for extrahepatic targeting	Human trial evidence (approved/clinically validated agents)
lncRNA (HOTTIP, MALAT1, HULC, SATB2-AS1, lincRNA-p21)	— (biomarkers/targets)	Regulation of proliferation, multidrug resistance, immune evasion	MALAT1 increases drug efflux; stable in blood/plasma/urine	Diagnostic/prognostic biomarkers for theranostic strategies	Clinical correlative evidence (biomarker studies; not therapeutic efficacy)
Drug/nucleic-acid co-delivery	LNPs, polymeric NPs, liposomes, hybrid nanocarriers	Synchronized stimuli-responsive release (pH, reductive environment, enzymatic activity)	Improved efficacy and reduced systemic toxicity with doxorubicin/cisplatin/paclitaxel + miRNA/siRNA	Stimuli-responsive NPs; SORT-LNPs; AI-assisted design	Animal efficacy (in vivo preclinical combinations)
Human serum albumin (HSA)	HSA-coated NPs	Endogenous transport: gp60 (transcytosis), FcRn (recycling), SPARC (stromal retention)	Partial reversal of P-glycoprotein-mediated multidrug resistance	Endogenous dual-function carrier (biocompatibility + active targeting)	Preclinical efficacy (in vitro/animal MDR reversal)
Cell-penetrating peptides (CPPs)	Direct conjugates; functionalized nanocarriers	Electrostatic membrane interaction; macropinocytosis	Increased antitumor activity with doxorubicin/methotrexate/paclitaxel; BBB crossing	pHLIP (pH-responsive), aCPP (MMP-activatable), hypoxia-responsive CPPs	Animal efficacy (in vivo preclinical, BBB crossing)
RGD/cRGD/iRGD	Peptide-functionalized NPs	αvβ3/αvβ5 integrin binding; iRGD binds neuropilin-1 (CendR motif)	Radiosensitization in glioblastoma; improved cisplatin/gefitinib delivery in lung cancer; enhanced immunotherapy in gastric cancer	AI-assisted discovery of new integrin-targeting peptides; CEND-1 (LSTA1) in phase I/II trials	Human trial evidence (CEND-1 in phase I/II); other constructs: animal efficacy (preclinical)

**Table 3:** Comparative analytical parameters of 2D monolayer and 3D in vitro models—spheroids, organoids, and organ-on-chip (OoC) systems.

Analytical Parameter	2D In Vitro Models (Monolayer)	3D Spheroids	3D Organoids	Organ-on-Chip
Cell Morphology	Flattened, artificial polarization forced by the rigid substrate [290]	Compact spherical aggregates; peripheral proliferating rim with a hypoxic/necrotic core in larger spheroids [291]	Complex, tissue-specific architecture (e.g., crypt-villus, ductal structures) self-organized from stem/progenitor cells [15]	Depends on device design; can host monolayers, spheroids, or organoid-like structures under dynamic flow within engineered microchannels [15]
Extracellular Matrix (ECM)	Practically absent; limited deposition on the bottom of the plate [290]	Self-secreted ECM only in scaffold-free aggregates; often minimal/disorganized unless embedded in exogenous matrix (e.g., Matrigel, collagen) [292]	Rich, physiologically organized ECM; self-organization typically requires embedding in basement-membrane matrices (e.g., Matrigel) [290,293]	Matrix composition and geometry are engineer-defined (synthetic hydrogels, collagen channels, membrane inserts); richness depends on device design rather than spontaneous assembly [15]
Physical-Chemical Gradients	Absent; uniform concentrations of O_2_, nutrients, and drugs [294]	Present; spontaneous radial gradients of hypoxia, pH, and nutrients that scale with spheroid diameter [295]	Present but variable; gradients depend on organoid size, budding architecture, and lumen formation, and are less predictable than in spheroids [15]	Engineered and controllable; continuous perfusion allows tunable, reproducible gradients (O_2_, shear stress, drug concentration) rather than purely passive diffusion [296]
Gene Expression Profiles	Distorted; stable alteration of integrins, receptors, and resistance genes [292]	Partially physiological; improved relative to 2D but can still diverge from in vivo tissue depending on cell line and culture duration [292]	Closest to physiological; stable transcriptional profiles similar to in vivo tissue, particularly for patient-derived organoids [15]	Physiological relevance strongly dependent on cell source and flow/mechanical conditioning; can approximate in vivo expression when properly optimized
Nanoparticle Accessibility	Direct, immediate, and uniform across the entire cell surface	Limited and size-dependent; diffusion barriers restrict penetration beyond the outer cell layers [294,297]	Limited and highly variable; luminal architecture and matrix embedding add penetration barriers beyond those seen in spheroids [15]	Governed by device geometry and flow; perfusable channels can improve delivery uniformity, but interstitial/matrix barriers persist in surrounding tissue compartments
False Positive/Negative Rate	High rate of false positives for efficacy; overestimation of cytotoxicity [294]	Reduced relative to 2D; may still underestimate efficacy if core necrosis/hypoxia is not accounted for [292]	Generally lower; better reproduction of in vivo drug-resistance mechanisms, though patient-derived variability affects consistency [15,292]	Potentially lowest among in vitro systems when vascular/immune components are included, but data remain limited by low sample numbers per experiment [15]
Cost and Operational Complexity	Minimal cost, standardized protocols, ideal for rapid HTS screening [291]	Moderate cost; relatively simple, scalable formation protocols (e.g., low-attachment plates, hanging drop) [291,298]	High cost; lengthy culture protocols requiring specialized matrices and growth-factor cocktails [292,298]	Highest cost and complexity; requires microfabrication, perfusion systems, and specialized imaging/analytics [15,292,298]
Reproducibility	High; well standardized across laboratories	Moderate; size and morphology vary with formation method (hanging-drop, low-attachment, microfluidic) [291]	Variable; strongly dependent on donor/patient source, passage number, and matrix lot [293]	Moderate-to-high for engineering parameters (flow, geometry), but biological reproducibility remains limited by cell-source variability [296]
Batch-to-Batch Variability	Low; established cell lines show minimal batch variability	Moderate; aggregate size and compactness can vary between production batches [291]	High; patient-/donor-derived material and basement-membrane matrix lot variability are major sources of inconsistency [293]	Moderate; device fabrication and hydrogel/matrix batch variability can affect performance, partially mitigated by standardized manufacturing [296]
Matrix Dependence	None; cells grow on rigid plastic/glass substrates without an embedding matrix	Optional; scaffold-free spheroids exist, but physiological relevance often requires embedding in exogenous matrix [292]	High; self-organization and long-term growth typically require embedding in basement-membrane matrices [293]	Variable and design-dependent; ranges from matrix-free perfused channels to fully matrix-embedded compartments [15]
Throughput	Very high; compatible with standard HTS/HCS multiwell formats [291]	Moderate-to-high; compatible with 96/384-well low-attachment formats and automated imaging [291]	Low-to-moderate; longer culture times and manual handling limit scale-up [15]	Low; inherently limited by device complexity and manual/microfluidic handling requirements [296]
Immune-Cell Inclusion	Absent by default; requires a separate co-culture setup	Possible via co-culture (e.g., tumor–immune cell spheroids), but not intrinsic to the base model	Increasingly incorporated in advanced co-culture/“assembloid” systems, but absent from standard organoid protocols [15]	Can be integrated directly via dedicated immune-cell perfusion channels, representing one of the platform’s key advantages [299]
Vascularization	Absent	Generally absent; vascularized spheroids require endothelial cell co-culture and remain non-perfused	Largely absent in standard protocols; vascularized organoids are an active but still emerging research area [15]	Can include perfusable, endothelial-lined microchannels mimicking vascular flow and shear stress, a distinguishing platform feature [296,300]

## Data Availability

No new data were created or analyzed in this study. Data sharing is not applicable to this article.

## Abbreviations

The following abbreviations are used in this manuscript:

AFP	alpha-fetoprotein
AgFON	silver film over nanospheres
AI	artificial intelligence
ALN	alendronate
ANNs	artificial neural networks
APTES	(3-Aminopropyl)triethoxysilane
APTMS	(3-Aminopropyl)trimethoxysilane
ASOs	antisense oligonucleotides
ATP	adenosine triphosphate
AuNS	gold nanostars
BBB	blood–brain barrier
BPNPs	biodegradable polymeric NPs
CARPA	complement activation-related pseudoallergy
CCR2	C-C Chemokine Receptor Type 2
CD	Cluster of Differentiation
CDK	Cyclin-Dependent Kinase
CEA	Carcinoembryonic Antigen
cGAS	Cyclic GMP-AMP Synthase
CH	chitosan
CMC	Chemistry, Manufacturing, and Controls
CNNs	convolutional neural networks
CPPs	critical process parameters
CTC	circulating tumor cells
CUR	curcumin
CXCR4	C-X-C Chemokine Receptor Type 4
DLS	Dynamic Light Scattering
DNA	deoxyribonucleic acid
DNN(s)	deep neural network(s)
DOX	doxorubicin
DPPH	2,2-Diphenyl-1-picrylhydrazyl
ECM	extracellular matrix
EGCG	epigallocatechin gallate
EGFR	Epidermal Growth Factor Receptor
EGFRvIII	Epidermal Growth Factor Receptor Variant III
EMA	European Medicines Agency
EpCAM	Epithelial Cell Adhesion Molecule
EPR	enhanced permeability and retention
ErbB2	Erythroblastic Oncogene B2 (HER2)
EVs	extracellular vesicles
FA	folic acid
FDA	Food and Drug Administration
FUS	focused ultrasound
GANs	generative adversarial networks
GLUT	Glucose Transporter
GMP	Good Manufacturing Practice
GNNs	graph neural networks
GNRs	gold nanorods
GSH	glutathione
HA	hyaluronic acid
HAase	hyaluronidase
HAp	hydroxyapatite
HCQ	hydroxychloroquine
HCS	High-Content Screening
HER2	Human Epidermal Growth Factor Receptor 2
HIF	Hypoxia-Inducible Factor
HPH	high-pressure homogenization
HSA	human serum albumin
HSP70	Heat Shock Protein 70
HTS	High-Throughput Screening
HUVEC	Human Umbilical Vein Endothelial Cell
HYAL1/HYAL2	hyaluronidase 1/2
IC50	half-maximal inhibitory concentration
ICD	immunogenic cell death
IDO	indoleamine 2,3-dioxygenase
IFP	interstitial fluid pressure
IL-10	interleukin-10
IRF3	Interferon Regulatory Factor 3
IVIS	in vivo imaging system
KRAS	Kirsten Rat Sarcoma Viral Oncogene Homolog
LBNPs	lipid-based NPs
LCA	Life Cycle Assessment
LNP(s)	lipid nanoparticle(s)
LSPR	localized surface plasmon resonance
LYC	lycopene
MAPK	mitogen-activated protein kinase
MCTSs	multicellular tumor spheroids
MD	molecular dynamics
MDR	multidrug resistance
MIRIBEL	Minimum Information Reporting in Bio–Nano Experimental Literature
MISEV2023	Minimal Information for Studies of Extracellular Vesicles 2023
miRNA	microRNA
ML	Machine Learning
MMP	matrix metalloproteinase
MOF(s)	metal–organic framework(s)
MPS	mononuclear phagocyte system
mRNA	messenger RNA
MRI	Magnetic Resonance Imaging
MS	Mass Spectrometry
MSNs	mesoporous silica NPs
NF-κB	Nuclear Factor Kappa-Light-Chain-Enhancer of Activated B Cells
NLCs	nanostructured lipid carriers
NIR	near-infrared
NKT	Natural Killer T (cell)
NPs	NPs
OoC	organ-on-a-chip
PA	photoacoustic
PAT	process analytical technologies
PBPK	physiologically-based pharmacokinetic
PCL	polycaprolactone
PD-1	Programmed Cell Death Protein 1
PD-L1	Programmed Death-Ligand 1
PDA	polydopamine
PDOs	patient-derived organoids
PDT	photodynamic therapy
PDX	Patient-Derived Xenograft
PEG	Polyethylene Glycol
P-gp	P-glycoprotein
PHEA	α,β-Poly(N-2-hydroxyethyl)-D,L-aspartamide
PI	propidium iodide
PLA	polylactic acid
PLGA	poly(lactic-co-glycolic acid)
PNIPAM	poly(N-isopropylacrylamide)
PTT	photothermal therapy
PVA	poly(vinyl alcohol)
PVP	polyvinylpyrrolidone
QbD	Quality by Design
QSAR	quantitative structure–activity relationship
RAFT	Reversible Addition–Fragmentation Chain Transfer
RBC	red blood cell (erythrocyte)
RGD	arginine-glycine-aspartate (peptide)
RHE	reconstructed human epidermis
RISC	RNA-Induced Silencing Complex
RNA	ribonucleic acid
ROS	reactive oxygen species
SbD	Safe-by-Design
SDL	self-driving laboratory
SEM	scanning electron microscopy
SERS	Surface-Enhanced Raman Spectroscopy/Scattering
SEDDS	Self-Emulsifying Drug Delivery Systems
SHAP	SHapley Additive exPlanations
siRNA	small interfering RNA
SIRPα	Signal Regulatory Protein Alpha
SLNs	solid lipid NPs
SORT	selective organ targeting
SPION(s)	superparamagnetic iron oxide nanoparticle(s)
STING	Stimulator of Interferon Genes
SWCNTs	single-walled carbon nanotubes
TAMs	tumor-associated macrophages
TBK1	TANK-Binding Kinase 1
TEM	transmission electron microscopy
TEXs	tumor-derived exosomes
TGF-β	Transforming Growth Factor Beta
THY	thymol
TLR	Toll-Like Receptor
TME	tumor microenvironment
TNBC	triple-negative breast cancer
TPP	Target Product Profile
UCNPs	upconversion NPs
UV	ultraviolet
VAEs	variational autoencoders
VEGF	Vascular Endothelial Growth Factor
WBC	white blood cell
XAI	explainable artificial intelligence
XGBoost	Extreme Gradient Boosting
ZIF-8	Zeolitic Imidazolate Framework-8
